# Patterns in Symptoms Preceding Acute Care in Patients With Cancer

**DOI:** 10.1001/jamanetworkopen.2025.6366

**Published:** 2025-04-22

**Authors:** Chichi Chang, Jie Jane Chen, Jean Feng, Isabel Friesner, Somya Mohindra, Lauren Boreta, Michael W. Rabow, Steve E. Braunstein, Ryzen Benson, Julian C. Hong

**Affiliations:** 1 Bakar Computational Health Sciences Institute, University of California, San Francisco; 2 Department of Radiation Oncology, University of California, San Francisco; 3 Department of Epidemiology and Biostatistics, University of California, San Francisco; 4 Division of Palliative Medicine, Department of Internal Medicine, University of California, San Francisco; 5 Department of Urology, University of California, San Francisco; 6 UCSF-UC Berkeley Joint Program in Computational Precision Health, San Francisco, California

## Abstract

**Importance:**

Patients with cancer frequently experience unplanned acute care with emergency department visits and hospitalization due to disease or treatment complications, which impacts outcomes, quality of life, and health care costs. There remains a knowledge gap in understanding patterns of symptoms that precede acute care events. Natural language processing (NLP) may enable greater understanding of the symptoms and identify differences across patient and cancer characteristics.

**Objective:**

To characterize symptoms preceding acute care in patients with cancer and quantify differences in symptom documentation across sociodemographic and cancer histologic subgroups.

**Design, Setting, and Participants:**

A cohort study in a single tertiary-care institution, including all acute care (emergency department and hospitalization) encounters for patients aged 18 years or older with a primary cancer diagnosis identified between January 1, 2013, and December 31, 2023.

**Main Outcomes and Measures:**

Natural language processing was used to identify routine clinical documentation to characterize symptoms documented in the 30 days preceding acute care. Logistic regression analyses was used to examine the possible association between sex, age, race and ethnicity, insurance coverage, cancer histologic characteristics, and reported symptoms.

**Results:**

Overall, 28 708 patients with cancer had 70 606 acute care visits with 854 830 associated preceding documented symptoms. Median age was 61 (IQR, 48-70) years. Men (37 861 encounters [53.62%]) and patients of White race (39 989 encounters [56.64%]) accounted for most acute care encounters. Pain (7.54% of documented symptoms), nausea (6.74%), and vomiting (5.79%) were the most frequently documented symptoms. Acute care encounters with patients who were female (adjusted odds ratio [AOR], 1.14; 95% CI, 1.10-1.18; *P* < .001), Asian (AOR, 1.22; 1.17-1.28; *P* < .001), Black (AOR, 1.17; 95% CI, 1.10-1.25; *P* < .001), American Indian or Alaska Native (AOR, 1.21; 95% CI, 1.01-1.44; *P* = .04), or Medicaid-insured (AOR, 1.10; 95% CI, 1.05-1.14; *P* < .001) were associated with a high documented symptom burden (>10 unique symptoms) preceding acute care visits. Patients aged 65 years or older (AOR, 0.96; 95% CI, 0.92-1.00; *P* = .04) or uninsured (AOR, 0.58; 95% CI, 0.45-0.76; *P* < .001) were less likely to have a high symptom burden documented before acute care events.

**Conclusions and Relevance:**

The findings of this study highlight common symptoms preceding acute care as well as the need for further research on interventions to reduce patient burden, improve quality of life, and reduce the use of acute care in patients with cancer.

## Key Points

**Question:**

What symptoms are most frequently documented preceding acute care visits (emergency department visits or hospitalizations) among patients with cancer and are they documented differently across sociodemographic and cancer characteristics?

**Findings:**

In a cohort study including 28 708 patients with cancer, pain, nausea, and vomiting were the most commonly documented symptoms. Encounters with patients who were female and Medicaid-insured were associated with a greater symptom burden, while patients who were of White race, aged 65 or older, or uninsured were less likely to have a high symptom burden documented preceding acute care events.

**Meaning:**

The findings of this study underscore frequently documented symptoms preceding acute care use and sociodemographic differences, suggesting the potential need to develop targeted interventions for improved symptom management.

## Teaser

This cohort study examines the symptoms documented preceding the use of emergency department visits and unplanned hospitalization in patients with cancer.

## Introduction

An estimated 2 million new cancer cases in the US are projected in 2025.^1^ Many of these patients will require unplanned acute care with an emergency department (ED) visit or hospital admission due to acute complications from the disease, symptoms from treatment, or comorbidities.^2,3,4^ Such unplanned acute care can greatly affect patient outcomes, quality of life, and health care system spending.^2,5,6^ Nationally, there was an estimated 67% increase in ED visits by patients with cancer from 2012 to 2019, of which more than 50% of the visits were potentially preventable.^7^ The Centers for Medicare & Medicaid Services has taken action to propose Measure OP-35 to reduce preventable acute care use among patients with cancer.^8,9,10,11^

Physical and psychological symptoms have been identified as primary reasons for unplanned acute care.^12,13^ Symptom management and identification of patients at high risk for unplanned visits are commonly proposed strategies and effective strategies to reduce acute hospital care.^5,14,15,16^ Studies conducted to date have primarily focused on characterizing patients with acute visit–related encounters through cancer diagnoses or documented symptoms within a homogeneous population, such as patients with shared cancer histologic factors (eg, breast cancer).^17,18,19,20,21,22,23,24^

Despite well-documented data on disparities in cancer care management and unmet symptom management needs, there remains a knowledge gap in understanding the sociodemographic differences in symptom identification and how they may potentially impact cancer care management.^25,26,27^ Previous studies have highlighted racial and ethnic disparities in patient-reported symptom burden among patients with cancer.^28,29,30^ Thus, early identification of key symptoms and patterns associated with ED visits and hospitalizations may lead to improvements in symptom management and clinical outcomes.^31,32,33^

In this study, we applied a previously validated natural language processing (NLP) pipeline to identify patterns of documented symptoms in the short term preceding ED visits and unplanned hospitalizations. We investigated the symptom burden of patients with cancer prior to acute care events and delineated potential sociodemographic patterns in the documentation of these symptoms. These findings may promote early patient-centered interventions.

## Methods

### Study Population

This study was approved by the University of California, San Francisco Institutional Review Board and waiver of informed consent was granted by the reviewing institutional board, given the use of deidentified data. We identified all adult patients aged 18 years or older with primary cancer diagnoses at our institution via a single tertiary-care institutional deidentified electronic health record (EHR) between January 1, 2013, and December 31, 2023. Patient diagnoses were identified based on *International Statistical Classification of Diseases and Related Health Problems, Tenth Revision* (*ICD-10*) and *International Classification of Diseases, Ninth Revision* (*ICD-9*) codes. Clinical data, clinical notes, and metadata on clinical notes for the patient cohort were extracted using computational code at scale from the EHR rather than manual retrieval. This cohort study followed the Strengthening the Reporting of Observational Studies in Epidemiology (STROBE) reporting guideline.

Acute care visits (ED visits and hospital admissions) were identified in this cohort (Figure). To ensure a sufficient history following a cancer diagnosis and preceding acute care events, only acute care visits at least 30 days after a documented cancer diagnosis were included in this study. Patients were excluded if their metadata or clinical notes were missing.

**Figure.**
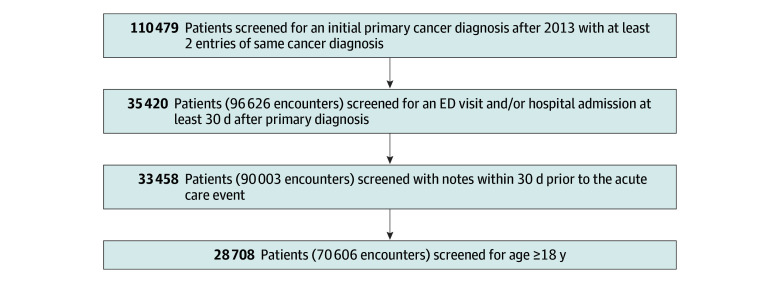
Patient Cohort Selection Between January 1, 2013, and December 31, 2023 ED indicates emergency department.

### Study Measures and Outcomes

We obtained patient self-reported sex, race and ethnicity, age, insurance coverage, and cancer diagnosis from the deidentified EHR, with categorizations derived from the EHR. These were collected as part of routine care. The Clinical Text Analysis and Knowledge Extraction System (cTAKES; Apache) is applied to all clinical notes at our institution. Briefly, this is an open-source, clinical text–centric NLP modular system combining rule-based and machine learning techniques.^34^ We then additionally applied a previously described pipeline to convert cTAKES SNOMED extracts into all Common Terminology Criteria for Adverse Events (CTCAE) version 5.0–encoded symptoms documented in all clinical notes in the 30 days preceding an ED visit or hospitalization.^35^ This overall approach has been externally validated against physician annotators, and the processing code is available on GitHub.^35^ All dates within the notes were shifted by a random patient-level offset and identifiable text was removed to maintain anonymity.^36^

The primary outcome was documented symptom burden and characterization of documented symptoms preceding acute care visits for patients with cancer. Secondary outcomes included examination of associations and comparison of symptoms across sociodemographic (sex, race and ethnicity, age, and insurance coverage) and cancer histologic factor subgroups. We used a previously described definition of symptom burden, which is defined as the quantifiable number of symptoms experienced by a patient specific to a medical illness at any point in time.^37,38^ Patients for a given acute care encounter were considered as experiencing a high documented symptom burden if there were more than 10 unique documented symptoms among all possible CTCAE symptoms recorded across all clinical notes in the 30 days before the acute care event.

### Statistical Analysis

We assessed the top NLP-extracted documented symptoms and age, sex, race and ethnicity, insurance coverage, and primary cancer diagnosis with univariable and multivariable logistic regression analyses. Encounters were only labeled with unspecified malignant neoplasm for their primary diagnosis if there was no other cancer diagnosis present, including metastasis. Patients with more than 1 primary cancer diagnosis were identified as having multiple cancer diagnoses. Logistic regression modeling was performed in Python Statsmodel package, version 0.12.1 (Python). To account for repeated acute care visits within patients, generalized estimating equations (GEEs) were performed with the geepack package in R, version 4.2.3 (R Foundation for Statistical Computing).^39,40^ All statistical tests were 2-tailed, with *P* ≤ .05 considered significant.

## Results

### Patient Cohort Characteristics

Sociodemographic and clinical characteristics are presented in Table 1. A total of 110 479 patients with cancer were identified between January 1, 2013, and December 31, 2023, of which 28 708 patients with an ED visit and/or hospital admission more than 30 days after the first documented diagnosis were included in our analysis. The median number of acute care visits per patient was 1 (IQR, 1-3). The median number of days between the first documented cancer diagnosis and acute care visit was 120 (IQR, 56-360) days. Of the 70 606 acute care encounters, 15 380 (53.57%) men accounted for 37 861 (53.62%) and 13 319 (46.39%) women accounted for 32 728 (46.35%). The median age at the first acute care encounter was 61 (IQR, 48-70) years. There were 12 477 encounters (17.67%) among 4414 (15.38%) Asian patients, 4905 (6.95%) among 1583 (5.51%) Black or African American patients, 533 (0.75%) among 225 (0.78%) American Indian or Alaska Native patients, 627 (0.89%) among 225 (0.78%) Native Hawaiian or Other Pacific Islander patients, 39 989 (56.64%) among 17 871 (62.26%) White patients, and 11 401 (16.15%) among 3948 (13.75%) patients with other race (no further information for other race is available). Private insurance was the most common, with 32 477 encounters (46.00%), followed by Medicare with 22 565 encounters (31.96%) and Medicaid with 14 181 encounters (20.08%). There were 69 349 (98.22%) encounters with a single cancer diagnosis, of which the most common known cancer diagnoses were hematologic in 15 183 encounters (21.50%), prostate in 4926 encounters (6.98%), and breast in 3624 encounters (5.13%). Patient characteristics are presented in eTable 1 in Supplement 1.

**Table 1.** Acute Care Encounter Patient Characteristics (N = 70 606)

Variables	No. (%)
No. of unique patients	28 708
Sex	
Male	37 861 (53.62)
Female	32 728 (46.35)
Unknown	17 (0.02)
Age, y	
Median (IQR)	61 (48-70)
18-64	42 528 (60.23)
≥65	28 078 (39.77)
Race and ethnicity^a^	
American Indian or Alaska Native	533 (0.75)
Asian	12 477 (17.67)
Black or African American	4905 (6.95)
Native Hawaiian or Other Pacific Islander	627 (0.89)
Unknown/declined	670 (0.95)
White	39 989 (56.64)
Other^b^	11 401 (16.15)
Insurance	
Private	32 477 (46.00)
Medicare	22 565 (31.96)
Medicaid	14 181 (20.08)
Uninsured/self-pay	268 (0.38)
Other	127 (0.18)
Unspecified	988 (1.40)
High symptom burden	
>10 Symptoms	35 998 (50.98)
Metastasis	14 905 (21.11)
Cancer diagnosis	
Unspecified malignant neoplasm	15 969 (22.62)
Hematologic	15 183 (21.50)
Prostate	4926 (6.98)
Breast	3624 (5.13)
Liver and bile duct	3524 (4.99)
Gynecologic	3008 (4.26)
CNS	2794 (3.96)
Nonmelanoma skin	2720 (3.85)
Colorectal	2691 (3.81)
Bone soft tissue and sarcoma	2680 (3.80)
Bladder and ureter	2119 (3.00)
Lung	1481 (2.10)
Head and neck	1299 (1.84)
Pancreatic	1260 (1.78)
Multiple diagnosis	1257 (1.78)
Kidney and pelvis of the kidney	1158 (1.64)
GI other	1044 (1.48)
Melanoma	516 (0.73)
Other	3353 (4.75)

Abbreviations: CNS, central nervous system; GI, gastrointestinal.

^a^ All categories were obtained from patient self-report in the electronic health record.

^b^ No further information is available for this category.

### Most Common Documented Symptoms and Symptom Burden

Natural language processing identified a total of 854 830 instances of 137 of the 837 symptoms in CTCAE version 5.0 (eTable 2 in Supplement 1) in the 30 days preceding the 70 606 acute care events. There was a median of 11 (IQR, 6-17) symptoms preceding each acute care event. The top 10 most common symptoms reported preceding an acute care visit were pain (7.54% of documented symptom presence before acute care), nausea (6.74%), vomiting (5.79%), fatigue (5.26%), constipation (3.93%), fever (3.39%), generalized muscle weakness (3.32%), extremity edema (3.28%), dyspnea (3.12%), and headache (2.92%). Documentation of more than 1 of these symptoms was possible in the period preceding each acute care encounter. A total of 35 998 encounters (50.98%) were considered as a high documented symptom burden (>10 symptoms reported).

In both univariable and multivariable analyses (Table 2), patients who were female (odds ratio [OR], 1.21; 95% CI, 1.17-1.24; *P* < .001; adjusted OR [AOR], 1.14; 95% CI, 1.10-1.18; *P* < .001), Asian (OR, 1.36; 95% CI, 1.31-1.42; *P* < .001; AOR, 1.22; 95% CI, 1.17-1.28; *P* < .001), Black race (OR, 1.22; 95% CI, 1.15-1.29; *P* < .001); AOR, 1.17; 95% CI, 1.10-1.25; *P* < .001), American Indian or Alaska Native race (OR, 1.26; 95% CI, 1.06-1.49; *P* = .009; AOR, 1.21; 95% CI, 1.01-1.44; *P* = .04) or Medicaid-insured (OR, 1.26; 95% CI, 1.21-1.32; *P* < .001; AOR, 1.10; 95% CI, 1.05-1.14; *P* < .001) were more likely to have a high documented symptom burden preceding an acute care visit. Compared with patients younger than 65 years and privately insured, those aged 65 years or older (OR, 0.82; 95% CI, 0.80-0.85; *P* < .001; AOR, 0.96; 95% CI, 0.92-1.00; *P* = .04) or uninsured (OR, 0.48; 95% CI, 0.37-0.62; *P* < .001; AOR, 0.58; 95% CI, 0.45-0.76; *P* < .001) were less likely to experience a high documented symptom burden. Prostate cancer had a lower rate of high documented symptom burden, with multiple cancer diagnoses (OR, 5.54; 95% CI, 4.85-6.32; *P* < .001; AOR, 4.83; 95% CI, 4.23-5.52; *P* < .001) or hematologic malignant neoplasm (OR, 5.41; 95% CI, 5.02-5.82; *P* < .001, AOR, 4.65; 95% CI, 4.31-5.02; *P* < .001) significantly associated with high documented symptom burden.

**Table 2.** Logistic Regression for Symptom Burden (>10 Symptoms) Across Demographic and Cancer Characteristics

Variable	Univariable			Multivariable	
	OR (95% CI)	*P* value	Estimated margin	OR (95% CI)	*P* value
Sex					
Male	1 [Reference]	NA	0.49	1 [Reference]	NA
Female	1.21 (1.17-1.24)	<.001	0.54	1.14 (1.10-1.18)	<.001
Unknown	0.73 (0.28-1.93)	.53	0.41	0.61 (0.23-1.63)	.32
Age, y					
18-64	1 [Reference]	NA	0.53	1 [Reference]	NA
≥65	0.82 (0.80-0.85)	<.001	0.48	0.96 (0.92-1.00)	.04
Race and ethnicity^a^					
American Indian or Alaska Native	1.26 (1.06-1.49)	.009	0.54	1.21 (1.01-1.44)	.04
Asian	1.36 (1.31-1.42)	<.001	0.56	1.22 (1.17-1.28)	<.001
Black or African American	1.22 (1.15-1.29)	<.001	0.53	1.17 (1.10-1.25)	<.001
Native Hawaiian or Other Pacific Islander	1.25 (1.07-1.47)	.005	0.54	1.16 (0.99-1.37)	.07
White	1 [Reference]	NA	0.48	1 [Reference]	NA
Unknown/declined	0.63 (0.54-0.74)	<.001	0.37	0.65 (0.55-0.76)	<.001
Other	1.34 (1.29-1.40)	<.001	0.55	1.13 (1.08-1.19)	<.001
Insurance					
Private	1 [Reference]	NA	0.51	1 [Reference]	NA
Medicaid	1.26 (1.21-1.32)	<.001	0.57	1.10 (1.05-1.14)	<.001
Medicare	0.90 (0.87-0.94)	<.001	0.48	0.96 (0.92-1.01)	.11
Other	0.92 (0.65-1.30)	.62	0.49	0.90 (0.63-1.29)	.56
Uninsured/self-pay	0.48 (0.37-0.62)	<.001	0.33	0.58 (0.45-0.76)	<.001
Unspecified	0.39 (0.34-0.45)	<.001	0.29	0.58 (0.51-0.68)	<.001
Cancer category					
Prostate	1 [Reference]	NA	0.23	1 [Reference]	NA
Bladder and ureter	2.64 (2.37-2.95)	<.001	0.44	2.50 (2.24-2.78)	<.001
Bone soft tissue and sarcoma	4.23 (3.82-4.68)	<.001	0.56	3.60 (3.25-3.99)	<.001
Breast	2.22 (2.02-2.44)	<.001	0.40	1.81 (1.64-2.00)	<.001
CNS	2.17 (1.97-2.40)	<.001	0.40	1.94 (1.75-2.15)	<.001
Colorectal	3.15 (2.85-3.48)	<.001	0.49	2.75 (2.48-3.05)	<.001
GI other	3.27 (2.85-3.75)	<.001	0.50	2.85 (2.48-3.28)	<.001
Gynecologic	2.81 (2.55-3.10)	<.001	0.46	2.23 (2.01-2.47)	<.001
Head and neck	3.64 (3.21-4.14)	<.001	0.52	3.23 (2.84-3.67)	<.001
Hematologic	5.41 (5.02-5.82)	<.001	0.62	4.65 (4.31-5.02)	<.001
Kidney and pelvis of the kidney	1.92 (1.68-2.21)	<.001	0.37	1.68 (1.46-1.93)	<.001
Liver and bile duct	3.04 (2.76-3.33)	<.001	0.48	2.60 (2.36-2.86)	<.001
Lung	3.61 (3.20-4.08)	<.001	0.52	3.13 (2.77-3.55)	<.001
Melanoma	2.20 (1.83-2.66)	<.001	0.40	2.05 (1.69-2.48)	<.001
Multiple diagnoses	5.54 (4.85-6.32)	<.001	0.63	4.83 (4.23-5.52)	<.001
Nonmelanoma skin	2.08 (1.88-2.30)	<.001	0.38	1.95 (1.76-2.16)	<.001
Other	2.19 (1.99-2.41)	<.001	0.40	1.89 (1.71-2.08)	<.001
Pancreatic	4.17 (3.67-4.75)	<.001	0.56	3.65 (3.20-4.16)	<.001
Unspecified malignant neoplasm	5.00 (4.65-5.38)	<.001	0.60	4.27 (3.96-4.61)	<.001

Abbreviations: CNS, central nervous system; GI, gastrointestinal; NA, not applicable; OR, odds ratio.

^a^ All categories were obtained from patient self-report in the electronic health record, and no further information is available for the category chosen as other.

### Symptom Documentation Across Patient Demographics

On univariable analysis (Table 3; eTables 3 and 4 in Supplement 1), women had increased rates of symptom documentation compared with men across all top 10 symptoms, including greater odds of documented nausea (OR, 1.26; 95% CI, 1.21-1.31; *P* < .001) or headache (OR, 1.33; 95% CI, 1.29-1.38; *P* < .001). Encounters with patients aged 65 years or older were associated with a decreased likelihood of documenting 9 of the top 10 most common symptoms, of which headache (OR, 0.57; 95% CI, 0.55-0.59; *P* < .001) and nausea (OR, 0.61; 95% CI, 0.59-0.64; *P* < .001) were the lowest. Patients of races and ethnicities other than White had an increased likelihood of reporting generalized muscle weakness, with the highest risk among Asian (OR, 1.40; 95% CI, 1.34-1.46; *P* < .001) or Black or African American (OR, 1.36; 95% CI, 1.28-1.44, *P* < .001) individuals.

**Table 3.** Univariable Regression Analysis for Documented Symptoms Across Demographic and Cancer Characteristics

Variable	Pain			Nausea			Vomiting		
	OR (95% CI)	*P* value	Estimated margin	OR (95% CI)	*P* value	Estimated margin	OR (95% CI)	*P* value	Estimated margin
Sex									
Male	1 [Reference]	NA	0.91	1 [Reference]	NA	0.80	1 [Reference]	NA	0.69
Female	1.20 (1.14-1.26)	<.001	0.92	1.12 (1.09-1.16)	<.001	0.83	1.15 (1.12-1.19)	<.001	0.72
Unknown	0.78 (0.18-3.40)	.74	0.88	1.45 (0.51-4.10)	.71	0.76	1.48 (0.48-4.54)	.49	0.76
Age, y									
18-64	1 [Reference]	NA	0.92	1 [Reference]	NA	0.85	1 [Reference]	NA	0.73
≥65	0.70 (0.66-0.73)	<.001	0.90	0.84 (0.81-0.86)	<.001	0.77	0.67 (0.65-0.69)	<.001	0.65
Race and ethnicity^a^									
American Indian or Alaska Native	1.27 (0.91-1.78)	.16	0.93	1.31 (1.09-1.57)	.89	0.82	1.07 (0.88-1.29)	.49	0.72
Asian	0.84 (0.79-0.90)	<.001	0.90	1.15 (1.10-1.20)	<.001	0.81	0.88 (0.84-0.91)	<.001	0.68
Black or African American	0.94 (0.85-1.04)	.24	0.91	0.90 (0.84-0.95)	<.001	0.75	0.70 (0.66-0.74)	<.001	0.63
Native Hawaiian or Other Pacific Islander	1.14 (0.85-1.54)	.38	0.92	1.32 (1.11-1.57)	.89	0.82	0.93 (0.79-1.11)	.42	0.69
White	1 [Reference]	NA	0.91	1 [Reference]	NA	0.82	1 [Reference]	NA	0.71
Unknown/declined	1.08 (0.82-1.43)	.59	0.92	0.73 (0.62-0.85)	.57	0.81	1.10 (0.93-1.31)	.26	0.73
Other	1.17 (1.08-1.26)	<.001	0.93	1.08 (1.04-1.14)	<.001	0.84	1.08 (1.04-1.14)	.001	0.73
Insurance									
Private	1 [Reference]	NA	0.92	1 [Reference]	NA	0.84	1 [Reference]	NA	0.73
Medicaid	1.21 (1.12-1.30)	<.001	0.93	0.98 (0.94-1.03)	.04	0.84	1.01 (0.97-1.06)	.65	0.73
Medicare	0.79 (0.74-0.84)	<.001	0.90	0.83 (0.80-0.86)	<.001	0.77	0.68 (0.66-0.71)	<.001	0.64
Other	1.19 (0.60-2.35)	.61	0.93	1.29 (0.88-1.89)	.80	0.83	1.46 (0.95-2.25)	.09	0.80
Uninsured/self-pay	0.72 (0.49-1.06)	.09	0.89	0.77 (0.61-0.99)	.07	0.80	1.00 (0.77-1.32)	.98	0.73
Unspecified	0.91 (0.73-1.13)	.39	0.91	0.56 (0.49-0.63)	<.001	0.77	0.95 (0.83-1.09)	.48	0.72
Cancer category									
Prostate	1 [Reference]	NA	0.95	1 [Reference]	NA	0.83	1 [Reference]	NA	0.79
Bladder and ureter	0.95 (0.76-1.19)	.68	0.94	1.33 (1.20-1.48)	<.001	0.79	0.68 (0.60-0.76)	<.001	0.72
Bone soft tissue and sarcoma	1.61 (1.26-2.04)	<.001	0.97	1.85 (1.68-2.04)	<.001	0.86	0.96 (0.85-1.07)	.44	0.78
Breast	0.70 (0.59-0.83)	<.001	0.92	1.24 (1.13-1.35)	.001	0.80	0.66 (0.60-0.73)	<.001	0.71
CNS	0.30 (0.25-0.35)	<.001	0.84	1.62 (1.47-1.78)	<.001	0.78	0.53 (0.48-0.59)	<.001	0.67
Colorectal	0.67 (0.55-0.80)	<.001	0.92	1.81 (1.64-2.00)	.01	0.80	0.71 (0.64-0.79)	<.001	0.73
GI other	0.83 (0.63-1.09)	.18	0.93	1.80 (1.57-2.07)	.05	0.85	0.95 (0.81-1.12)	.57	0.78
Gynecologic	1.27 (1.02-1.57)	.03	0.96	1.67 (1.52-1.83)	<.001	0.88	1.15 (1.02-1.29)	.02	0.81
Head and neck	1.08 (0.82-1.42)	.59	0.95	1.35 (1.20-1.53)	.23	0.84	0.80 (0.69-0.93)	.003	0.75
Hematologic	0.38 (0.33-0.43)	<.001	0.87	2.07 (1.94-2.21)	.45	0.83	0.47 (0.44-0.51)	<.001	0.64
Kidney and pelvis of the kidney	0.72 (0.56-0.93)	.01	0.93	1.14 (1.00-1.30)	<.001	0.75	0.52 (0.45-0.60)	<.001	0.66
Liver and bile duct	0.38 (0.33-0.45)	<.001	0.87	1.41 (1.30-1.54)	<.001	0.74	0.40 (0.36-0.44)	<.001	0.60
Lung	0.58 (0.47-0.72)	<.001	0.91	1.42 (1.26-1.60)	<.001	0.75	0.47 (0.41-0.53)	<.001	0.64
Melanoma	0.79 (0.55-1.14)	.21	0.93	1.02 (0.85-1.23)	<.001	0.75	0.50 (0.41-0.61)	<.001	0.66
Multiple diagnoses	0.63 (0.50-0.79)	<.001	0.92	1.85 (1.63-2.11)	.09	0.81	0.55 (0.48-0.63)	<.001	0.68
Nonmelanoma skin	0.34 (0.29-0.40)	<.001	0.86	0.81 (0.74-0.89)	<.001	0.60	0.25 (0.22-0.27)	<.001	0.48
Other	0.94 (0.78-1.14)	.53	0.94	1.37 (1.25-1.50)	.78	0.83	0.79 (0.71-0.87)	<.001	0.75
Pancreatic	1.14 (0.85-1.51)	.38	0.95	1.91 (1.68-2.17)	<.001	0.90	1.18 (1.01-1.38)	.04	0.82
Unspecified malignant neoplasm	0.88 (0.77-1.01)	.08	0.94	2.42 (2.27-2.59)	<.001	0.85	0.71 (0.66-0.77)	<.001	0.73

Abbreviations: CNS, central nervous system; GI, gastrointestinal; NA, not applicable; OR, odds ratio.

^a^ All categories were obtained from patient self-report in the electronic health record, and no further information is available for the category chosen as other.

On multivariable analysis (Table 4; eTables 5 and 6 in Supplement 1), age 65 years or older (AOR, 1.18, 95% CI, 1.13-1.24; *P* < .001) or Black or African American race (AOR, 1.42; 95% CI, 1.33-1.51; *P* < .001) were independently associated with documentation of generalized muscle weakness. There was decreased documentation of nausea preceding an acute care visit among adults aged 65 years or older (AOR, 0.71; 95% CI, 0.67-0.74; *P* < .001), Asian race (AOR, 0.83; 95% CI, 0.79-0.88; *P* < .001), or Black or African American race (AOR, 0.57; 95% CI, 0.53-0.61; *P* < .001).

**Table 4.** Multivariable Regression Analysis for Documented Symptoms Across Demographic and Cancer Characteristics

Variable	Pain		Nausea		Vomiting	
	OR (95% CI)	*P* value	OR (95% CI)	*P* value	OR	*P* value
Sex						
Male	1 [Reference]	NA	1 [Reference]	NA	1 [Reference]	NA
Female	1.15 (1.09-1.22)	<.001	1.27 (1.21-1.32)	<.001	1.15 (1.11-1.20)	<.001
Unknown	0.84 (0.19-3.75)	.82	0.73 (0.23-2.30)	.59	1.50 (0.48-4.71)	.48
Age, y						
18-64	1 [Reference]	NA	1 [Reference]	NA	1 [Reference]	NA
≥65	0.72 (0.67-0.77)	<.001	0.71 (0.67-0.74)	<.001	0.74 (0.71-0.78)	<.001
Race and ethnicity^a^						
American Indian or Alaska Native	1.02 (0.72-1.43)	.93	0.83 (0.66-1.04)	.10	0.90 (0.74-1.09)	.27
Asian	0.81 (0.75-0.87)	<.001	0.83 (0.79-0.88)	<.001	0.84 (0.80-0.88)	<.001
Black or African American	0.78 (0.70-0.87)	<.001	0.57 (0.53-0.61)	<.001	0.60 (0.57-0.64)	<.001
Native Hawaiian or Other Pacific Islander	0.98 (0.72-1.32)	.88	0.84 (0.69-1.04)	.11	0.82 (0.69-0.97)	.02
White	1 [Reference]	NA	1 [Reference]	NA	1 [Reference]	NA
Unknown/declined	0.96 (0.72-1.27)	.77	0.85 (0.70-1.04)	.12	0.99 (0.83-1.17)	.87
Other	1.13 (1.04-1.22)	.005	1.03 (0.97-1.09)	.41	1.03 (0.98-1.08)	.28
Insurance						
Private	1 [Reference]	NA	1 [Reference]	NA	1 [Reference]	NA
Medicaid	1.18 (1.09-1.28)	<.001	0.87 (0.83-0.93)	<.001	1.00 (0.95-1.05)	>.99
Medicare	0.94 (0.87-1.01)	.09	0.80 (0.76-0.85)	<.001	0.83 (0.79-0.87)	<.001
Other	1.20 (0.61-2.39)	.60	0.92 (0.57-1.48)	.73	1.45 (0.94-2.25)	.10
Uninsured/self-pay	0.74 (0.50-1.09)	.13	0.78 (0.58-1.06)	.11	0.94 (0.72-1.24)	.67
Unspecified	0.76 (0.61-0.96)	.02	0.63 (0.54-0.73)	<.001	0.82 (0.71-0.94)	.006
Cancer category						
Prostate	1 [Reference]	NA	1 [Reference]	NA	1 [Reference]	NA
Bladder and ureter	0.93 (0.74-1.16)	.52	0.74 (0.65-0.84)	<.001	0.67 (0.59-0.75)	<.001
Bone soft tissue and sarcoma	1.27 (1.00-1.62)	.05	1.06 (0.93-1.22)	.39	0.78 (0.70-0.88)	<.001
Breast	0.53 (0.44-0.64)	<.001	0.56 (0.50-0.63)	<.001	0.49 (0.44-0.55)	<.001
CNS	0.23 (0.19-0.27)	<.001	0.54 (0.48-0.61)	<.001	0.41 (0.36-0.45)	<.001
Colorectal	0.55 (0.46-0.67)	<.001	0.70 (0.62-0.79)	<.001	0.60 (0.54-0.67)	<.001
GI other	0.75 (0.57-0.99)	.05	1.06 (0.88-1.28)	.55	0.87 (0.74-1.02)	.09
Gynecologic	0.92 (0.74-1.15)	.48	1.07 (0.92-1.23)	.38	0.87 (0.77-0.98)	.02
Head and neck	0.98 (0.74-1.29)	.88	1.01 (0.85-1.19)	.94	0.75 (0.65-0.87)	<.001
Hematologic	0.30 (0.26-0.34)	<.001	0.80 (0.73-0.88)	<.001	0.38 (0.35-0.41)	<.001
Kidney and pelvis of the kidney	0.64 (0.49-0.82)	<.001	0.56 (0.48-0.65)	<.001	0.46 (0.40-0.54)	<.001
Liver and bile duct	0.32 (0.28-0.38)	<.001	0.49 (0.44-0.55)	<.001	0.34 (0.31-0.38)	<.001
Lung	0.56 (0.45-0.70)	<.001	0.60 (0.52-0.69)	<.001	0.46 (0.40-0.52)	<.001
Melanoma	0.65 (0.45-0.93)	.02	0.46 (0.37-0.57)	<.001	0.39 (0.32-0.48)	<.001
Multiple diagnoses	0.55 (0.43-0.69)	<.001	0.76 (0.65-0.89)	.001	0.50 (0.43-0.57)	<.001
Nonmelanoma skin	0.31 (0.26-0.36)	<.001	0.26 (0.23-0.29)	<.001	0.21 (0.19-0.24)	<.001
Other	0.74 (0.61-0.90)	.003	0.75 (0.66-0.84)	<.001	0.62 (0.55-0.69)	<.001
Pancreatic	1.02 (0.77-1.36)	.87	1.56 (1.28-1.90)	<.001	1.07 (0.91-1.25)	.44
Unspecified malignant neoplasm	0.72 (0.63-0.84)	<.001	0.94 (0.86-1.03)	.18	0.59 (0.54-0.64)	<.001

Abbreviations: CNS, central nervous system; GI, gastrointestinal; NA, not applicable; OR, odds ratio.

^a^ All categories were obtained from patient self-report in the electronic health record, and no further information is available for the category chosen as other.

### Symptom Documentation by Insurance Coverage

Encounters for Medicaid-insured patients in comparison with privately insured patients were more likely to be associated with documented fever (OR, 1.34; 95% CI, 1.29-1.40; *P* < .001) or generalized muscle weakness (OR, 1.26; 95% CI, 1.21-1.31; *P* < .001) in the univariable analysis. Meanwhile, encounters for patients who were uninsured or self-pay were associated with a significantly decreased likelihood of reporting 7 of the top 10 symptoms, of which generalized muscle weakness (OR, 0.53; 95% CI, 0.40-0.70; *P* < .001) and constipation (OR, 0.58; 95% CI, 0.45-0.75; *P* < .001) were the lowest.

On multivariable analysis, patients who were Medicaid-insured were more likely to have documented pain (OR, 1.18; 95% CI, 1.09-1.28; *P* < .001) or generalized muscle weakness (OR, 1.14; 95% CI, 1.10-1.19; *P* < .001). Medicare-insured patients were more likely to have documented generalized muscle weakness (OR, 1.07; 95% CI, 1.02-1.12; *P* = .003) or dyspnea (OR, 1.06; 95% CI, 1.02-1.11; *P* = .01), but less likely to have documentation of all other symptoms, with the lowest odds of documented nausea (OR, 0.80; 95% CI, 0.76-0.85; *P* < .001). Uninsured or self-pay individuals were less likely to have documentation of headache (OR, 0.54; 95% CI, 0.41-0.72; *P* < .001) or generalized muscle weakness (OR, 0.58; 95% CI, 0.43-0.77; *P* < .001).

### Symptom Documentation by Cancer Histologic Factors

On univariable analysis, all cancer histologic factors in comparison with prostate cancer were associated with documented constipation, generalized muscle weakness, fever, edema, or dyspnea. Fever was documented among most cancer histologic factors, with the highest odds of documentation among patients with hematologic malignant neoplasms (OR, 6.76; 95% CI, 6.24-7.32; *P* < .001). Vomiting was less likely to be documented among most cancer histologic factors, with the lowest likelihood in encounters for nonmelanoma skin malignant neoplasms (OR, 0.25; 95% CI, 0.22-0.27; *P* < .001).

After adjusting for socioeconomic and clinical covariates, similar patterns of increased documentation of generalized muscle weakness among patients with hematologic cancer (AOR, 5.91; 95% CI, 5.42-6.45; *P* < .001) or central nervous system malignant neoplasms (AOR, 6.39; 95% CI, 5.73-7.13; *P* < .001) were found. Similarly, headache (AOR, 7.14; 95% CI, 6.40-7.97; *P* < .001) was also more frequently documented in encounters for patients with central nervous system malignant neoplasms. In the multivariate analysis, pain or vomiting were less likely to be documented in most cancer histologic factors, with nonmelanoma skin cancer presenting the lowest likelihood of documented vomiting compared with prostate cancer (AOR, 0.21; 95% CI, 0.19-0.24; *P* < .001).

### Symptom Burden and Documentation Results With GEEs 

Generalized estimating equations overall verified the limited impact on the results in patients with repeat acute care encounters. With regard to high documented symptom burden, GEEs did not dramatically alter ORs, although certain race and ethnicity–, age-, and insurance-related variables were not significant (including Medicaid insurance) (eTable 7 in Supplement 1). On multivariable analysis, high documented symptom burden was associated with sex and multiple race and ethnicity categories other than White. Similarly, fewer characteristics were associated across various symptoms (eTables 8 and 9 in Supplement 1).

## Discussion

In this study, we identified patterns and differences in symptom burden by examining NLP-extracted symptoms documented in clinical notes prior to acute care visits. We found that pain and nausea were the most frequently reported symptoms and patients with cancer who were women, of a race other than White, aged younger than 65 years, or Medicaid-insured had a greater documented symptom burden in the 30 days preceding an acute care visit. These findings may be grounded in the various upstream points of differential symptom manifestation, patient reporting, or health care professional documentation, and underscore the need to characterize potentially undertreated symptoms, which are often factors associated with worse clinical outcomes.^25,41,42,43^ To our knowledge, our study is the first to build on the findings of symptom characterization and highlight potential disparities seen in previous studies through a longitudinal lens.^26,44,45^ This gap calls for further analyses of reasons underlying these patterns and implementation of patient-centered interventions that can better serve potential unmet needs.

We found that women were more likely to experience a high documented symptom burden before a visit to the ED or hospitalization in comparison with men. This finding is consistent with prior studies, which have described inadequate management and underreporting of symptoms experienced by women.^46,47,48,49,50,51,52^ It may therefore be beneficial to incorporate systematic questions that screen for symptom severity levels or level of function impacted by symptoms during routine oncologic care to encourage symptom documentation among women. Further analyses are also needed to understand how symptoms potentially manifest differently in men and women. If differences arise, this insight can provide guidance for changes in the diagnostic workflow, specifically incorporating symptom-severity questions during visits that are sex-specific for improved symptom documentation and reduced symptom burden.

We also found that patients who were Medicaid-insured were more likely to experience a high documented symptom burden before acute care visits. Insurance coverage has previously been associated with disparities in cancer care, the stage at which cancer is diagnosed, and overall survival rates.^43,53,54,55^ Possible explanations for this increased prevalence in symptom burden can be attributed to limited access to care or lower cancer screening rates.^43,56^ Limited financial resources may deter patients from seeking early care, thus resulting in manifestations of unattended symptoms. However, whether these observed patterns may be more reflective of more complex histologic factors at advanced cancer stages, medical care avoidance due to high costs, or limited access requires further exploration. Inadequate resources and access to quality care remain barriers that can be overcome through policy changes, such as oncology social work service expansion or increased health care coverage. Studies have reported how integrating a social work component into outpatient cancer care contributes to clinically meaningful improvements of patients’ quality of life.^57,58^ Meanwhile, other studies have documented how Medicaid expansion is associated with improved cancer prognosis outcomes and survival in states with expanded Medicaid access compared with those without. This highlights how expanded health care coverage holds may have utility in reducing cost barriers that may contribute to delayed health care–seeking behaviors.^59^

Furthermore, younger people in our cohort had an increased risk of higher symptom burden documentation. These findings reiterate existing findings in the literature describing age-related disparities in symptom burden.^60^ One plausible hypothesis for this observation is that older adults may receive a lower dose of chemotherapy or radiotherapy due to fear of toxic effects and adverse effects.^61,62^ Conversely, younger patients may receive more aggressive treatments, which carry with them greater risks for toxic effects and therefore a greater consequent symptom burden. Another theory could be that younger adults experience much more chaotic lives, juggling responsibilities such as work, raising a family, and managing relationships. A study reported that younger adult patients with cancer with children described higher levels of pain and financial distress compared with those without children.^63^ This suggests that it is possible that distress from personal lives contributes to the increased symptom burden observed in younger adults. These speculations warrant investigation in future studies. One prospective approach to mitigate these differences in symptom burden is integrating routine screening for patients’ emotional and social distress in addition to physical distress into outpatient care, such as with the National Comprehensive Cancer Network Distress Thermometer.^64^ This strategy can help clinicians better understand patient stressors that encompass both patient- and diagnosis-specific elements so that we can design patient-specific interventions, such as specialized psychosocial services, to address their unique unmet social or medical needs.

Similarities in findings between the logistic regression and GEE analyses suggest that the impact of repeated visits across individual patients on the findings is minimal and highlights the robustness of the logistic regression results presented. However, it is worth highlighting that there were a few differences in the 2 modeling results. Specifically, there was a reduced number of statistically significant findings in the GEE analysis. For example, while Medicaid-insured patients were significantly more likely to experience a high documented symptom burden in the logistic regression analysis, this association was no longer observed after adjusting for multiple visits in the GEE model. This suggests that some of the findings in the ordinary model may be influenced by patients with repeated visits. While this adjusted analysis is important to consider, the information drawn from repeat encounters for the same patient should not necessarily be simply considered redundant.

### Limitations

Limitations of this study include that it was a retrospective review from a single institution. This impacts the potential completeness of data capture, with regard to symptom identification as well as acute care visits themselves. Patients identified in this cohort were predominantly White and insured, which may not fully capture a fully diverse population.^65^

The use of NLP allows evaluation at large scale, but limitations in the technology in addition to the inherent complexities of clinical documentation, such as copy-forwarded text, incomplete sentences, challenges in chronicity, contradictory documentation, and incomplete context in individual notes are all potential sources of inaccuracy.^66^ This may be a cause for our high rate of symptom detection in the 30 days preceding acute care encounters. It is worth highlighting among these that our institution routinely screens for and documents pain, which likely increases its prevalence as the most frequently documented symptom; for instance, pain documented as a 1 of a scale of 10 would still represent some degree of pain but otherwise would not be captured in a different setting. We note though, that our approach has been previously validated against physician abstractors, but even in those cases these limitations in documentation can impact consistency of physician-based symptom identification.^35^ In the future, we anticipate that it is likely that transformer-based large language models are likely to have more flexible performance to offset some of these concerns, but their validation against physician annotation across different use-cases in oncology remains a work in progress.^67,68^ It is also important to highlight that as interest increases in training large language models on health care data, documentation biases are critical to create accurate and robust algorithms.

Additionally, our analysis of studying the association by encounters can be impacted by overlapping symptoms for individual patients with multiple visits in short time frames and skewed representations across certain clinical variables. For example, encounters with uninsured patients (0.38% of total) are less likely to have a high documented symptom burden compared with those with privately insured patients (46.00% of total). This raises the question of whether this finding is generalizable or based on a few uninsured patients in this cohort. For the latter, the GEE findings suggest that repeated events have a limited impact on our overall results. Future work may incorporate multicenter patient cohorts to capture a more equal distribution across race and insurance coverage.

Another limitation lies in our symptom categorization approach with CTCAE, as the inherent complexity of CTCAE may lead to interrater variability issues.^69^ This remains a challenge for interpretability, as grouping certain symptoms into a category (eg, nausea and vomiting) could change the interpretability of our results. Nevertheless, CTCAE is a commonly used standard for oncologic toxic effects encoding and grading for standardization.

We observed a high percentage of encounters with patients documented as having a nonspecific malignant neoplasm. It is possible that inaccuracies in *ICD* coding in the EHR are responsible for this, although a prior study suggested that *ICD* coding is at least specific for metastasis.^70^ Because of this, we did not include it as an area of focus in investigating symptom differences. Some studies have suggested high clinical volume and frequent interruptions are potential barriers to recording accurate and complete patient care information in acute care settings; there is a pressing need for improved communication between continuing care physicians and ED physicians to promote better documentation.^71,72,73,74^

The identification of symptoms preceding acute care events and sociodemographic differences raises areas of potential disparities for further investigation. This may be an opportunity for clinicians to implement interventions and strategies for reducing symptom burden and improving cancer care across sociodemographic backgrounds. As individual and societal factors may impact the observed patterns, there is a need for targeted interventions based on patient needs. Research has described how strategies such as cancer-related self-efficacy home-care nursing programs along with technological and educational interventions for symptom management have the potential to improve health behaviors and reduce symptom burden across racial groups and cancer types.^75,76,77,78^ For example, studies have reported how greater physical activity and self-efficacy have been associated with a lower symptom burden, as well as better functional, emotional, and social well-being among patients with cancer.^75,79,80^ Another study has reported how a prechemotherapy education intervention may help decrease distress and improve physical outcomes.^81^

## Conclusions

To our knowledge, this is the first analysis to characterize NLP-extracted documented symptoms preceding acute care visits and their association with specific sociodemographic and clinical variables with a large dataset of patient records that span over a decade. This analysis highlights differences in cancer symptom documentation across racial, sex, and socioeconomic subgroups, suggesting potential areas of disparities. This raises attention to the potential need to develop targeted interventions to ensure equitable access to health care for improved symptom management.

## Notes

Supplement 1. **eTable 1.** Acute Care Encounter Patient Characteristics by Patient (N = 28 708) **eTable 2.** Mapping of MedDRA Codes to All CTCAE Symptoms (n = 837) Present in Patient Cohort Notes **eTable 3.** Univariable Analysis of Fatigue and Constipation Documentation Across Patient Demographics **eTable 4.** Univariable Analysis of Fever, Weakness, Edema, Dyspnea, and Headache Documentation Across Patient Demographics **eTable 5.** Multivariable Analysis of Fatigue and Constipation Documentation Across Patient Demographics **eTable 6.** Multivariable Analysis of Fever, Weakness, Edema, Dyspnea, and Headache Documentation Across Patient Demographics **eTable 7.** GEE Analysis for Symptom Burden Across Demographic and Cancer Characteristics **eTable 8.** Univariable GEE Analysis for Documented Symptoms Across Demographic and Cancer Characteristics **eTable 9.** Multivariable GEE Analysis for Documented Symptoms Across Demographic and Cancer Characteristics

Supplement 2.
Data Sharing Statement
