# Supplementary material for: Patterns in Symptoms Preceding Acute Care in Patients With Cancer
Source: JAMA Netw Open. 2025 Apr 22;8(4):e256366. doi: 10.1001/jamanetworkopen.2025.6366 (PMC12015675; doi:10.1001/jamanetworkopen.2025.6366)
Supplement: Supplement 1. — eTable 1. Acute Care Encounter Patient Characteristics by Patient (N = 28 708) eTable 2. Mapping of MedDRA Codes to All CTCAE Symptoms (n = 837) Present in Patient Cohort Notes eTable 3. Univariable Analysis of Fatigue and Constipation Documentation Across Patient Demographics eTable 4. Univariable Analysis of Fever, Weakness, Edema, Dyspnea, and Headache Documentation Across Patient Demographics eTable 5. Multivariable Analysis of Fatigue and Constipation Documentation Across Patient Demographics eTable 6. Multivariable Analysis of Fever, Weakness, Edema, Dyspnea, and Headache Documentation Across Patient Demographics eTable 7. GEE Analysis for Symptom Burden Across Demographic and Cancer Characteristics eTable 8. Univariable GEE Analysis for Documented Symptoms Across Demographic and Cancer Characteristics eTable 9. Multivariable GEE Analysis for Documented Symptoms Across Demographic and Cancer Characteristics [file jamanetwopen-e256366-s001.pdf]

Supplementary Online Content

Chang C, Chen JJ, Feng J, et al. Patterns in symptoms preceding acute care in patients with cancer. *JAMA Netw Open*. 2025;8(4):e256366. doi:10.1001/jamanetworkopen.2025.6366

eTable 1. Acute Care Encounter Patient Characteristics by Patient (N = 28,708)

eTable 2. Mapping of MedDRA Codes to All CTCAE Symptoms (n = 837) Present in Patient Cohort Notes

eTable 3. Univariable Analysis of Fatigue and Constipation Documentation Across Patient Demographics

eTable 4. Univariable Analysis of Fever, Weakness, Edema, Dyspnea, and Headache Documentation Across Patient Demographics

eTable 5. Multivariable Analysis of Fatigue and Constipation Documentation Across Patient Demographics

eTable 6. Multivariable Analysis of Fever, Weakness, Edema, Dyspnea, and Headache Documentation Across Patient Demographics

eTable 7. GEE Analysis for Symptom Burden Across Demographic and Cancer Characteristics

eTable 8. Univariable GEE Analysis for Documented Symptoms Across Demographic and Cancer Characteristics

eTable 9. Multivariable GEE Analysis for Documented Symptoms Across Demographic and Cancer Characteristics

This supplementary material has been provided by the authors to give readers additional information about their work.

© 2025 Chang C et al. *JAMA Network Open.*

eTable 1: Acute Care Encounter Patient Characteristics by Patient (N = 28,708)

Variables	N (%)
**Sex**	
Male	15,380 (53.57%)
Female	13,319 (46.39%)
Unknown	9 (0.03%)
**Age**	
Median	61
IQR	48-70
18-65	16,718 (58.23%)
>= 65	11,990 (41.77%)
**Race and Ethnicity**	
Asian	4,414 (15.38%)
Black or African American	1,583 (5.51%)
Native American or Alaska Native	225 (0.78%)
Native Hawaiian or Other Pacific Islander	225 (0.78%)
Unknown/Declined	442 (1.54%)
White or Caucasian	17,871 (62.26%)
Other	3,948 (13.75%)
**Insurance**	
Private	13,425 (46.76%)
Medicare	9,710 (33.82%)
Medicaid	4,596 (16.01%)
Uninsured/Self-Pay	185 (0.64%)
Other	73 (0.25%)
Unspecified	719 (2.50%)
**High Symptom Burden**	9,206 (32.07%)
**Metastasis**	4,459 (15.53%)
**Cancer Diagnosis**	
Unspecified malignant neoplasm	6,118 (21.31%)
Hematologic	3,832 (13.35%)
Prostate	3,515 (12.24%)
Breast	1,968 (6.86%)
Gynecologic	1,511 (5.26%)
Central nervous system	1,363 (4.75%)
Liver and Bile Duct	1,283 (4.47%)
Non-melanoma skin	1,187 (4.13%)
Colorectal	1,103 (3.84%)
Bladder and ureter	930 (3.24%)
Bone soft tissue and sarcoma	856 (2.98%)
Head and Neck	664 (2.31%)
Lung	640 (2.23%)
Kidney and renal pelvis	578 (2.01%)
Pancreatic	525 (1.83%)
GI other	464 (1.62%)
Melanoma	269 (0.94%)
Multiple Diagnosis	58 (0.20%)
Other	1,844 (6.42%)

© 2025 Chang C et al. *JAMA Network Open.*

eTable 2: Mapping of MedDRA Codes to All CTCAE Symptoms (n = 837) Present in Patient Cohort Notes

MedDRA Code	CTCAE Term	Symptom Presence in Notes
10033371	Pain	64444
10028813	Nausea	57636
10047700	Vomiting	49483
10016256	Fatigue	44942
10010774	Constipation	33623
10016558	Fever	28953
10062572	Generalized muscle weakness	28421
10050068	Edema limbs	28065
10013963	Dyspnea	26711
10019211	Headache	24931
10041349	Somnolence	23726
10016825	Flushing	22078
10000081	Abdominal pain	21563
10013573	Dizziness	21212
10003988	Back pain	21204
10025482	Malaise	20499
10012727	Diarrhea	18213
10003598	Atelectasis	14987
10008531	Chills	14110
10024264	Lethargy	14057
10011224	Cough	13865
10047924	Wheezing	13090
10022437	Insomnia	12638
10033557	Palpitations	10992
10003239	Arthralgia	10576
10021097	Hypotension	10102
10044565	Tremor	9555
10028411	Myalgia	9349
10028653	Myositis	9349
10037087	Pruritus	9154
10028836	Neck pain	9022
10013990	Dysuria	8732
10013786	Dry skin	7376
10016173	Fall	7080
10031009	Oral pain	7026
10017577	Gait disturbance	5763
10006002	Bone pain	5011
10046543	Urinary incontinence	4984
10021143	Hypoxia	4758
10042772	Syncope	4505
10001497	Agitation	4491
10040741	Sinus bradycardia	4300
10028130	Mucositis oral	4296
10028735	Nasal congestion	3918
10016750	Flank pain	3713
10038743	Restlessness	3270
10036790	Productive cough	3241
10022998	Irritability	3190

© 2025 Chang C et al. *JAMA Network Open.*

10013946	Dyspepsia	3171
10003504	Aspiration	3062
10013911	Dysgeusia	2983
10048038	Wound infection	2880
10034879	Phlebitis	2829
10068319	Oropharyngeal pain	2726
10000486	Acidosis	2639
10013781	Dry mouth	2616
10046555	Urinary retention	2421
10034263	Pelvic pain	2402
10037400	Pulmonary hypertension	2343
10037032	Proteinuria	2066
10033425	Pain in extremity	1908
10034960	Photophobia	1762
10014020	Ear pain	1694
10035623	Pleuritic pain	1536
10042241	Stridor	1536
10047340	Vertigo	1528
10020407	Hot flashes	1407
10020039	Hiccups	1329
10040865	Skin hyperpigmentation	1284
10036653	Presyncope	1250
10003662	Atrial flutter	1176
10036402	Postnasal drip	1124
10015958	Eye pain	1120
10016766	Flatulence	1025
10002653	Anosmia	1014
10046901	Vaginal discharge	966
10048994	Bladder spasm	961
10016059	Facial pain	915
10042458	Suicidal ideation	882
10061103	Dermatitis radiation	864
10006298	Breast pain	856
10048031	Wound dehiscence	765
10024378	Leukocytosis	764
10044055	Toothache	752
10031264	Osteonecrosis	734
10023215	Joint effusion	712
10003591	Ataxia	682
10029223	Neuralgia	673
10027313	Menorrhagia	669
10011703	Cyanosis	667
10014950	Eosinophilia	619
10041232	Sneezing	606
10011655	Cushingoid	561
10019705	Hepatic pain	532
10033474	Pain of skin	471
10021113	Hypothermia	442
10038695	Respiratory failure	434
10037868	Rash maculo-papular	432
10043345	Testicular pain	419

© 2025 Chang C et al. *JAMA Network Open.*

10034310	Penile pain	402
10039757	Scrotal pain	366
10013941	Dyspareunia	269
10024419	Libido decreased	268
10061339	Perineal pain	242
10019245	Hearing impaired	165
10039020	Rhabdomyolysis	143
10028154	Multi-organ failure	142
10019663	Hepatic failure	123
10018286	Gingival pain	113
10041103	Small intestinal perforation	111
10001540	Akathisia	102
10002953	Aphonia	89
10022095	Injection site reaction	85
10012218	Delirium	76
10024561	Lip pain	73
10038419	Renal colic	69
10020642	Hyperhidrosis	57
10038073	Rectal perforation	57
10020112	Hirsutism	55
10062501	Non-cardiac chest pain	50
10046809	Uterine pain	48
10004665	Biliary fistula	45
10025182	Lymph node pain	43
10040102	Seroma	34
10007739	Cataract	26
10002176	Anal stenosis	24
10051792	Infusion related reaction	23
10021305	Ileal perforation	21
10002156	Anal fistula	19
10007515	Cardiac arrest	17
10033314	Ovulation pain	16
10040400	Serum sickness	14
10062225	Urinary tract pain	14
10059639	Laryngeal obstruction	5
10024421	Libido increased	5
10012205	Delayed puberty	4
10065879	Gastrointestinal anastomotic leak	2
10023030	Ischemia cerebrovascular	0
10020667	Hyperlipidemia	0
10020670	Hypermagnesemia	0
10022402	INR increased	0
10020647	Hyperkalemia	0
10020680	Hypernatremia	0
10020639	Hyperglycemia	0
10027175	Memory impairment	0
10014383	Electrocardiogram QT corrected interval prolonged	0
10024382	Leukoencephalopathy	0
10061212	Hypoglossal nerve disorder	0
10022763	Intracranial hemorrhage	0

© 2025 Chang C et al. *JAMA Network Open.*

10061185	Glossopharyngeal nerve disorder	0
10020949	Hypocalcemia	0
10020943	Hypoalbuminemia	0
10015832	Extrapyramidal disorder	0
10051272	Facial muscle weakness	0
10061457	Facial nerve disorder	0
10020907	Hyperuricemia	0
10020870	Hypertriglyceridemia	0
10018767	Guillain-Barre syndrome	0
10050380	Electrocardiogram T wave abnormal	0
10020712	Hyperphosphatemia	0
10056910	GGT increased	0
10016987	Forced expiratory volume decreased	0
10016596	Fibrinogen decreased	0
10020508	Hydrocephalus	0
10020765	Hypersomnia	0
10027198	Meningismus	0
10020587	Hypercalcemia	0
10002646	Anorexia	0
10052426	Glucose intolerance	0
10061928	Radiculitis	0
10034580	Peripheral motor neuropathy	0
10034620	Peripheral sensory neuropathy	0
10059895	Urine output decreased	0
10056238	Phantom pain	0
10043770	Thyroid stimulating hormone increased	0
10063636	Pyramidal tract syndrome	0
10038130	Recurrent laryngeal nerve palsy	0
10050528	Ejection fraction decreased	0
10063761	Reversible posterior leukoencephalopathy syndrome	0
10039906	Seizure	0
10011368	Creatinine increased	0
10011268	CPK increased	0
10041416	Spasticity	0
10041549	Spinal cord compression	0
10033987	Paresthesia	0
10056388	Olfactory nerve disorder	0
10047580	Vital capacity abnormal	0
10053661	Oculomotor nerve disorder	0
10029864	Nystagmus	0
10047896	Weight gain	0
10047900	Weight loss	0
10029205	Nervous system disorders - Other, specify	0
10049182	White blood cell decreased	0
10062646	Pancreatic enzymes decreased	0
10001598	Alcohol intolerance	0
10001680	Alkalosis	0
10028417	Myasthenia gravis	0
10065794	Muscle weakness right-sided	0
10065780	Muscle weakness left-sided	0

© 2025 Chang C et al. *JAMA Network Open.*

10028041	Movements involuntary	0
10012174	Dehydration	0
10014625	Encephalopathy	0
10029366	Neutrophil count decreased	0
10014217	Edema cerebral	0
10028533	Myelodysplastic syndrome	0
10031282	Osteoporosis	0
10024574	Lipase increased	0
10065799	Fibrosis deep connective tissue	0
10065793	Pelvic soft tissue necrosis	0
10039226	Rotator cuff injury	0
10039722	Scoliosis	0
10065777	Soft tissue necrosis lower limb	0
10015688	Exostosis	0
10040139	Serum amylase increased	0
10065778	Soft tissue necrosis upper limb	0
10008496	Chest wall pain	0
10065798	Superficial soft tissue fibrosis	0
10044684	Trismus	0
10048831	Chest wall necrosis	0
10065738	Unequal limb length	0
10064658	Osteonecrosis of jaw	0
10018761	Growth suppression	0
10065779	Head soft tissue necrosis	0
10028395	Musculoskeletal and connective tissue disorder - Other, specify	0
10065776	Muscle weakness lower limb	0
10024842	Lordosis	0
10065795	Muscle weakness trunk	0
10022891	Investigations - Other, specify	0
10065895	Muscle weakness upper limb	0
10055599	Hemoglobin increased	0
10023509	Kyphosis	0
10065781	Neck soft tissue necrosis	0
10065783	Musculoskeletal deformity	0
10065800	Joint range of motion decreased lumbar spine	0
10019150	Haptoglobin decreased	0
10042244	Stroke	0
10065796	Joint range of motion decreased cervical spine	0
10048706	Joint range of motion decreased	0
10048293	Leukemia secondary to oncology chemotherapy	0
10029104	Neoplasms benign, malignant and unspecified (incl cysts and polyps) - Other, specify	0
10013951	Dysphasia	0
10040907	Skin papilloma	0
10003074	Arachnoiditis	0
10021059	Hypophosphatemia	0
10065417	Brachial plexopathy	0
10065784	Central nervous system necrosis	0
10008164	Cerebrospinal fluid leakage	0

© 2025 Chang C et al. *JAMA Network Open.*

10009845	Cognitive disturbance	0
10010250	Concentration impairment	0
10012373	Depressed level of consciousness	0
10021038	Hyponatremia	0
10021028	Hypomagnesemia	0
10021018	Hypokalemia	0
10013887	Dysarthria	0
10028294	Muscle cramp	0
10062872	Dysesthesia	0
10021005	Hypoglycemia	0
10065973	Iron overload	0
10027433	Metabolism and nutrition disorders - Other, specify	0
10029883	Obesity	0
10066480	Avascular necrosis	0
10049737	Treatment related secondary malignancy	0
10055351	Tumor hemorrhage	0
10045158	Tumor pain	0
10053662	Abducens nerve disorder	0
10048677	Buttock pain	0
10025256	Lymphocyte count decreased	0
10003246	Arthritis	0
10045152	Tumor lysis syndrome	0
10060929	Accessory nerve disorder	0
10025258	Lymphocyte count increased	0
10018748	Growth hormone abnormal	0
10000521	Acoustic nerve disorder NOS	0
10065775	Abdominal soft tissue necrosis	0
10001949	Amnesia	0
10035528	Platelet count decreased	0
10002272	Anemia	0
10043245	Tendon reflex decreased	0
10040747	Sinus pain	0
10035759	Pneumothorax	0
10037375	Pulmonary edema	0
10037383	Pulmonary fibrosis	0
10065873	Pulmonary fistula	0
10038738	Respiratory, thoracic and mediastinal disorders - Other, specify	0
10038921	Retinoic acid syndrome	0
10039100	Rhinorrhea	0
10062244	Sinus disorder	0
10040975	Sleep apnea	0
10015218	Erythema multiforme	0
10041367	Sore throat	0
10065787	Tracheal fistula	0
10065900	Tracheal mucositis	0
10050816	Tracheal stenosis	0
10047681	Voice alteration	0
10001760	Alopecia	0
10005901	Body odor	0
10006556	Bullous dermatitis	0

© 2025 Chang C et al. *JAMA Network Open.*

10035742	Pneumonitis	0
10055319	Pleural hemorrhage	0
10035598	Pleural effusion	0
10034844	Pharyngolaryngeal pain	0
10051228	Chylothorax	0
10015090	Epistaxis	0
10020201	Hoarseness	0
10023838	Laryngeal edema	0
10065786	Laryngeal fistula	0
10065759	Laryngeal hemorrhage	0
10065735	Laryngeal inflammation	0
10065880	Laryngeal mucositis	0
10023862	Laryngeal stenosis	0
10062667	Laryngopharyngeal dysesthesia	0
10023891	Laryngospasm	0
10056356	Mediastinal hemorrhage	0
10034825	Pharyngeal fistula	0
10055315	Pharyngeal hemorrhage	0
10065881	Pharyngeal mucositis	0
10065706	Pharyngeal necrosis	0
10050028	Pharyngeal stenosis	0
10014184	Eczema	0
10015277	Erythroderma	0
10044391	Transient ischemic attacks	0
10019428	Hematoma	0
10042344	Subcutaneous emphysema	0
10043189	Telangiectasia	0
10044223	Toxic epidermal necrolysis	0
10046735	Urticaria	0
10041244	Social circumstances - Other, specify	0
10042613	Surgical and medical procedures - Other, specify	0
10073529	Arterial thromboembolism	0
10007196	Capillary leak syndrome	0
10020772	Hypertension	0
10016241	Fat atrophy	0
10065773	Lymph leakage	0
10025233	Lymphedema	0
10048642	Lymphocele	0
10034578	Peripheral ischemia	0
10042554	Superficial thrombophlebitis	0
10042569	Superior vena cava syndrome	0
10043565	Thromboembolic event	0
10047065	Vascular disorders - Other, specify	0
10042033	Stevens-Johnson syndrome	0
10040947	Skin ulceration	0
10051837	Skin induration	0
10040868	Skin hypopigmentation	0
10055525	Hair color changes	0
10019049	Hair texture abnormal	0
10020649	Hyperkeratosis	0

© 2025 Chang C et al. *JAMA Network Open.*

10020864	Hypertrichosis	0
10021013	Hypohidrosis	0
10062315	Lipohypertrophy	0
10028689	Nail changes	0
10028691	Nail discoloration	0
10049281	Nail loss	0
10062283	Nail ridging	0
10054524	Palmar-plantar erythrodysesthesia syndrome	0
10034966	Photosensitivity	0
10037549	Purpura	0
10037847	Rash acneiform	0
10049120	Scalp pain	0
10040785	Skin and subcutaneous tissue disorders - Other, specify	0
10040799	Skin atrophy	0
10006482	Bronchospasm	0
10065746	Bronchopulmonary hemorrhage	0
10053481	Bronchopleural fistula	0
10029164	Nephrotic syndrome	0
10069339	Acute kidney injury	0
10063575	Bladder perforation	0
10064848	Chronic kidney disease	0
10063057	Cystitis noninfective	0
10007839	CD4 lymphocytes decreased	0
10068405	Glucosuria	0
10019450	Hematuria	0
10019489	Hemoglobinuria	0
10038359	Renal and urinary disorders - Other, specify	0
10063524	Bronchial stricture	0
10038385	Renal calculi	0
10038463	Renal hemorrhage	0
10065368	Urinary fistula	0
10046539	Urinary frequency	0
10061574	Urinary tract obstruction	0
10046593	Urinary urgency	0
10046628	Urine discoloration	0
10001927	Amenorrhea	0
10042464	Suicide attempt	0
10037234	Psychosis	0
10037175	Psychiatric disorders - Other, specify	0
10034719	Personality change	0
10060890	Trigeminal nerve disorder	0
10074765	Trochlear nerve disorder	0
10061403	Vagus nerve disorder	0
10047166	Vasovagal reaction	0
10054746	Fetal growth retardation	0
10072314	Pregnancy loss	0
10036585	Pregnancy, puerperium and perinatal conditions - Other, specify	0
10036595	Premature delivery	0
10002652	Anorgasmia	0
10002855	Anxiety	0

© 2025 Chang C et al. *JAMA Network Open.*

10010300	Confusion	0
10057066	Delayed orgasm	0
10012260	Delusions	0
10012378	Depression	0
10015533	Euphoria	0
10019077	Hallucinations	0
10026749	Mania	0
10003883	Azoospermia	0
10006179	Breast atrophy	0
10013934	Dysmenorrhea	0
10065805	Spermatic cord obstruction	0
10055347	Testicular hemorrhage	0
10065811	Uterine fistula	0
10046789	Uterine hemorrhage	0
10065928	Uterine obstruction	0
10046904	Vaginal dryness	0
10065813	Vaginal fistula	0
10046912	Vaginal hemorrhage	0
10046916	Vaginal inflammation	0
10065817	Vaginal obstruction	0
10046937	Vaginal pain	0
10065818	Vaginal perforation	0
10053496	Vaginal stricture	0
10001409	Adult respiratory distress syndrome	0
10001723	Allergic rhinitis	0
10002972	Apnea	0
10006437	Bronchial fistula	0
10006440	Bronchial obstruction	0
10043306	Testicular disorder	0
10065762	Spermatic cord hemorrhage	0
10014326	Ejaculation disorder	0
10038604	Reproductive system and breast disorders - Other, specify	0
10061461	Erectile dysfunction	0
10065789	Fallopian tube obstruction	0
10054382	Feminization acquired	0
10018146	Genital edema	0
10018801	Gynecomastia	0
10060602	Hematosalpinx	0
10022992	Irregular menstruation	0
10061261	Lactation disorder	0
10065823	Nipple deformity	0
10030300	Oligospermia	0
10065763	Ovarian hemorrhage	0
10033279	Ovarian rupture	0
10064026	Pelvic floor muscle weakness	0
10036601	Premature menopause	0
10055325	Prostatic hemorrhage	0
10055026	Prostatic obstruction	0
10036968	Prostatic pain	0
10008661	Cholesterol high	0

© 2025 Chang C et al. *JAMA Network Open.*

10065815	Urethral anastomotic leak	0
10007613	Cardiac troponin T increased	0
10023176	Jejunal stenosis	0
10017815	Gastric perforation	0
10061970	Gastric stenosis	0
10017822	Gastric ulcer	0
10017853	Gastritis	0
10066874	Gastroesophageal reflux disease	0
10017947	Gastrointestinal disorders - Other, specify	0
10017877	Gastrointestinal fistula	0
10017999	Gastrointestinal pain	0
10018043	Gastroparesis	0
10060640	Hemorrhoidal hemorrhage	0
10019611	Hemorrhoids	0
10065728	Ileal fistula	0
10055287	Ileal hemorrhage	0
10065730	Ileal obstruction	0
10021307	Ileal stenosis	0
10021309	Ileal ulcer	0
10021328	Ileus	0
10055291	Intra-abdominal hemorrhage	0
10065719	Jejunal fistula	0
10055300	Jejunal hemorrhage	0
10065732	Jejunal obstruction	0
10051886	Gastric necrosis	0
10017789	Gastric hemorrhage	0
10065713	Gastric fistula	0
10062570	Enterovesical fistula	0
10012318	Dental caries	0
10013828	Duodenal fistula	0
10055242	Duodenal hemorrhage	0
10013830	Duodenal obstruction	0
10013832	Duodenal perforation	0
10050094	Duodenal stenosis	0
10013836	Duodenal ulcer	0
10013950	Dysphagia	0
10014893	Enterocolitis	0
10065851	Esophageal fistula	0
10016296	Fecal incontinence	0
10015384	Esophageal hemorrhage	0
10065727	Esophageal necrosis	0
10015387	Esophageal obstruction	0
10015388	Esophageal pain	0
10055472	Esophageal perforation	0
10015448	Esophageal stenosis	0
10015451	Esophageal ulcer	0
10015453	Esophageal varices hemorrhage	0
10015461	Esophagitis	0
10023174	Jejunal perforation	0
10023177	Jejunal ulcer	0
10042435	Sudden death NOS	0

© 2025 Chang C et al. *JAMA Network Open.*

10051746	Lower gastrointestinal hemorrhage	0
10039411	Salivary gland fistula	0
10065710	Small intestinal mucositis	0
10041101	Small intestinal obstruction	0
10062263	Small intestinal stenosis	0
10041133	Small intestine ulcer	0
10042112	Stomach pain	0
10044030	Tooth development disorder	0
10044031	Tooth discoloration	0
10045271	Typhlitis	0
10055356	Upper gastrointestinal hemorrhage	0
10054692	Visceral arterial ischemia	0
10011912	Death neonatal	0
10011914	Death NOS	0
10061818	Disease progression	0
10014222	Edema face	0
10058720	Edema trunk	0
10016791	Flu like symptoms	0
10018065	General disorders and administration site conditions - Other, specify	0
10018112	Generalized edema	0
10064774	Infusion site extravasation	0
10062466	Localized edema	0
10056681	Salivary duct inflammation	0
10038981	Retroperitoneal hemorrhage	0
10038080	Rectal ulcer	0
10033645	Pancreatitis	0
10025476	Malabsorption	0
10029957	Obstruction gastric	0
10065720	Oral cavity fistula	0
10054520	Oral dysesthesia	0
10030980	Oral hemorrhage	0
10065703	Pancreatic duct stenosis	0
10049192	Pancreatic fistula	0
10033626	Pancreatic hemorrhage	0
10058096	Pancreatic necrosis	0
10034536	Periodontal disease	0
10038079	Rectal stenosis	0
10065704	Peritoneal necrosis	0
10036774	Proctitis	0
10064993	Rectal fissure	0
10038062	Rectal fistula	0
10038064	Rectal hemorrhage	0
10063190	Rectal mucositis	0
10065709	Rectal necrosis	0
10065707	Rectal obstruction	0
10038072	Rectal pain	0
10010006	Colonic ulcer	0
10010004	Colonic stenosis	0
10010001	Colonic perforation	0
10010000	Colonic obstruction	0

© 2025 Chang C et al. *JAMA Network Open.*

10053565	Pericardial tamponade	0
10034484	Pericarditis	0
10061541	Pulmonary valve disease	0
10038748	Restrictive cardiomyopathy	0
10058597	Right ventricular dysfunction	0
10040639	Sick sinus syndrome	0
10040752	Sinus tachycardia	0
10042604	Supraventricular tachycardia	0
10061389	Tricuspid valve disease	0
10047281	Ventricular arrhythmia	0
10047290	Ventricular fibrillation	0
10047302	Ventricular tachycardia	0
10010331	Congenital, familial and genetic disorders - Other, specify	0
10013993	Ear and labyrinth disorders - Other, specify	0
10065785	External ear pain	0
10065838	Middle ear inflammation	0
10043882	Tinnitus	0
10047386	Vestibular disorder	0
10001367	Adrenal insufficiency	0
10014698	Endocrine disorders - Other, specify	0
10018746	Growth accelerated	0
10034474	Pericardial effusion	0
10034040	Paroxysmal atrial tachycardia	0
10028606	Myocarditis	0
10003658	Atrial fibrillation	0
10048580	Bone marrow hypocellular	0
10013442	Disseminated intravascular coagulation	0
10016288	Febrile neutropenia	0
10019491	Hemolysis	0
10019515	Hemolytic uremic syndrome	0
10027506	Methemoglobinemia	0
10043648	Thrombotic thrombocytopenic purpura	0
10061589	Aortic valve disease	0
10003586	Asystole	0
10003673	Atrioventricular block complete	0
10028596	Myocardial infarction	0
10003674	Atrioventricular block first degree	0
10007541	Cardiac disorders - Other, specify	0
10008481	Chest pain - cardiac	0
10010276	Conduction disorder	0
10019279	Heart failure	0
10069501	Left ventricular systolic dysfunction	0
10061532	Mitral valve disease	0
10027786	Mobitz (type) II atrioventricular block	0
10027787	Mobitz type I	0
10020705	Hyperparathyroidism	0
10020850	Hyperthyroidism	0
10021041	Hypoparathyroidism	0
10002167	Anal pain	0
10046851	Uveitis	0

© 2025 Chang C et al. *JAMA Network Open.*

10047516	Vision decreased	0
10047656	Vitreous hemorrhage	0
10047848	Watering eyes	0
10000060	Abdominal distension	0
10002153	Anal fissure	0
10055226	Anal hemorrhage	0
10065721	Anal mucositis	0
10065722	Anal necrosis	0
10002180	Anal ulcer	0
10038923	Retinopathy	0
10003445	Ascites	0
10004222	Belching	0
10005265	Bloating	0
10065747	Cecal hemorrhage	0
10008417	Cheilitis	0
10009167	Chylous ascites	0
10009887	Colitis	0
10009995	Colonic fistula	0
10009998	Colonic hemorrhage	0
10061510	Scleral disorder	0
10038901	Retinal vascular disorder	0
10062767	Hypophysitis	0
10015919	Eye disorders - Other, specify	0
10021067	Hypopituitarism	0
10021114	Hypothyroidism	0
10058084	Precocious puberty	0
10067734	Testosterone deficiency	0
10047488	Virilization	0
10005886	Blurred vision	0
10048492	Corneal ulcer	0
10013774	Dry eye	0
10015829	Extraocular muscle paresis	0
10061145	Eyelid function disorder	0
10038897	Retinal tear	0
10016757	Flashing lights	0
10016778	Floaters	0
10018304	Glaucoma	0
10023332	Keratitis	0
10029404	Night blindness	0
10061322	Optic nerve disorder	0
10033703	Papilledema	0
10054541	Periorbital edema	0
10038848	Retinal detachment	0
10054482	Neck edema	0
10069480	Vaccination site lymphadenopathy	0
10007612	Cardiac troponin I increased	0
10065847	Intraoperative splenic injury	0
10022213	Injury to inferior vena cava	0
10065849	Injury to jugular vein	0
10022356	Injury to superior vena cava	0

© 2025 Chang C et al. *JAMA Network Open.*

10022117	Injury, poisoning and procedural complications - Other, specify	0
10059095	Intestinal stoma leak	0
10059094	Intestinal stoma obstruction	0
10049468	Intestinal stoma site bleeding	0
10065826	Intraoperative arterial injury	0
10065831	Intraoperative breast injury	0
10065843	Intraoperative cardiac injury	0
10065844	Intraoperative ear injury	0
10065834	Intraoperative endocrine injury	0
10065825	Intraoperative gastrointestinal injury	0
10065842	Intraoperative head and neck injury	0
10055298	Intraoperative hemorrhage	0
10065827	Intraoperative hepatobiliary injury	0
10065829	Intraoperative musculoskeletal injury	0
10065830	Intraoperative neurological injury	0
10065841	Intraoperative ocular injury	0
10065845	Intraoperative renal injury	0
10065840	Intraoperative reproductive tract injury	0
10022161	Injury to carotid artery	0
10020100	Hip fracture	0
10065712	Gastrointestinal stoma necrosis	0
10002544	Ankle fracture	0
10048762	Tooth infection	0
10044302	Tracheitis	0
10046300	Upper respiratory infection	0
10052298	Urethral infection	0
10046571	Urinary tract infection	0
10062233	Uterine infection	0
10046914	Vaginal infection	0
10054688	Viremia	0
10065772	Vulval infection	0
10002899	Aortic injury	0
10065893	Gastric anastomotic leak	0
10003162	Arterial injury	0
10050458	Biliary anastomotic leak	0
10065802	Bladder anastomotic leak	0
10006504	Bruising	0
10006634	Burn	0
10065961	Esophageal anastomotic leak	0
10065788	Fallopian tube anastomotic leak	0
10065790	Fallopian tube perforation	0
10017076	Fracture	0
10065832	Intraoperative respiratory injury	0
10065828	Intraoperative urinary injury	0
10051341	Bile duct stenosis	0
10065848	Intraoperative venous injury	0
10065886	Uterine anastomotic leak	0
10046810	Uterine perforation	0
10046861	Vaccination complication	0
10065887	Vaginal anastomotic leak	0

10065888	Vas deferens anastomotic leak	0
10062169	Vascular access complication	0
10047228	Venous injury	0
10053692	Wound complication	0
10048049	Wrist fracture	0
10000636	Activated partial thromboplastin time prolonged	0
10001551	Alanine aminotransferase increased	0
10001675	Alkaline phosphatase increased	0
10003481	Aspartate aminotransferase increased	0
10005332	Blood antidiuretic hormone abnormal	0
10005359	Blood bicarbonate decreased	0
10005364	Blood bilirubin increased	0
10005452	Blood corticotrophin decreased	0
10005561	Blood gonadotrophin abnormal	0
10005630	Blood lactate dehydrogenase increased	0
10005778	Blood prolactin abnormal	0
10065906	Carbon monoxide diffusing capacity decreased	0
10065885	Urostomy stenosis	0
10065748	Urostomy site bleeding	0
10065883	Urostomy obstruction	0
10065894	Rectal anastomotic leak	0
10065803	Kidney anastomotic leak	0
10065891	Large intestinal anastomotic leak	0
10050457	Pancreatic anastomotic leak	0
10065705	Pharyngeal anastomotic leak	0
10055322	Postoperative hemorrhage	0
10056745	Postoperative thoracic procedure complication	0
10065745	Prolapse of intestinal stoma	0
10065822	Prolapse of urostomy	0
10037767	Radiation recall reaction (dermatologic)	0
10065892	Small intestinal anastomotic leak	0
10065882	Urostomy leak	0
10065897	Spermatic cord anastomotic leak	0
10041569	Spinal fracture	0
10065898	Stenosis of gastrointestinal stoma	0
10042127	Stomal ulcer	0
10062548	Tracheal hemorrhage	0
10044291	Tracheal obstruction	0
10065749	Tracheostomy site bleeding	0
10065814	Ureteric anastomotic leak	0
10005329	Blood and lymphatic system disorders - Other, specify	0
10043649	Thrush	0
10064505	Stoma site infection	0
10062112	Splenic infection	0
10062255	Soft tissue infection	0
10003999	Bacteremia	0
10061695	Biliary tract infection	0
10005047	Bladder infection	0
10061017	Bone infection	0

© 2025 Chang C et al. *JAMA Network Open.*

10006259	Breast infection	0
10055078	Bronchial infection	0
10007810	Catheter related infection	0
10065761	Cecal infection	0
10008330	Cervicitis infection	0
10010741	Conjunctivitis	0
10010742	Conjunctivitis infective	0
10061788	Corneal infection	0
10065765	Cranial nerve infection	0
10058666	Cytomegalovirus infection reactivation	0
10064687	Device related infection	0
10065752	Duodenal infection	0
10014594	Encephalitis infection	0
10014621	Encephalomyelitis infection	0
10014678	Endocarditis infective	0
10014801	Endophthalmitis	0
10058838	Enterocolitis infectious	0
10065744	Arteritis infective	0
10003012	Appendicitis perforated	0
10003011	Appendicitis	0
10019805	Hepatobiliary disorders - Other, specify	0
10006537	Budd-Chiari syndrome	0
10008612	Cholecystitis	0
10017631	Gallbladder fistula	0
10059446	Gallbladder necrosis	0
10017636	Gallbladder obstruction	0
10017638	Gallbladder pain	0
10017639	Gallbladder perforation	0
10019678	Hepatic hemorrhage	0
10019692	Hepatic necrosis	0
10034405	Perforation bile duct	0
10061640	Anorectal infection	0
10036200	Portal hypertension	0
10036206	Portal vein thrombosis	0
10063675	Sinusoidal obstruction syndrome	0
10001718	Allergic reaction	0
10002218	Anaphylaxis	0
10061664	Autoimmune disorder	0
10052015	Cytokine release syndrome	0
10021428	Immune system disorders - Other, specify	0
10056519	Abdominal infection	0
10015109	Epstein-Barr virus infection reactivation	0
10058804	Esophageal infection	0
10015929	Eye infection	0
10061351	Pleural infection	0
10069138	Papulopustular rash	0
10034016	Paronychia	0
10058674	Pelvic infection	0
10061912	Penile infection	0
10051472	Periorbital infection	0
10065766	Peripheral nerve infection	0

© 2025 Chang C et al. *JAMA Network Open.*

10057262	Peritoneal infection	0
10034835	Pharyngitis	0
10056627	Phlebitis infective	0
10050662	Prostate infection	0
10055005	Ovarian infection	0
10037888	Rash pustular	0
10059827	Rhinitis infective	0
10039413	Salivary gland infection	0
10062156	Scrotal infection	0
10040047	Sepsis	0
10040555	Shingles	0
10040753	Sinusitis	0
10040872	Skin infection	0
10065771	Small intestine infection	0
10051741	Pancreas infection	0
10033078	Otitis media	0
10016936	Folliculitis	0
10023216	Joint infection	0
10017544	Fungemia	0
10062632	Gallbladder infection	0
10018784	Gum infection	0
10056522	Hepatic infection	0
10058827	Hepatitis B reactivation	0
10019799	Hepatitis viral	0
10080137	Herpes simplex reactivation	0
10021881	Infections and infestations - Other, specify	0
10021918	Infective myositis	0
10023424	Kidney infection	0
10033072	Otitis externa	0
10023874	Laryngitis	0
10065755	Lip infection	0
10061229	Lung infection	0
10050823	Lymph gland infection	0
10057483	Mediastinal infection	0
10027199	Meningitis	0
10065764	Mucosal infection	0
10028524	Myelitis	0
10061304	Nail infection	0
10047115	Vasculitis	0

© 2025 Chang C et al. *JAMA Network Open.*

eTable 3. Univariable Analysis of Fatigue and Constipation Documentation Across Patient Demographics

Variables	Fatigue			Constipation		
	OR	p-value	Predicted Margins	OR	p-value	Predicted Margins
**Sex**						
Male	Ref		0.62	Ref		0.45
Female	1.12 (1.09-1.16)	<.001	0.65	1.25 (1.21-1.29)	<.001	0.51
Unknown	1.45 (0.51-4.10)	0.49	0.71	0.85 (0.32-2.24)	0.75	0.41
**Age**						
18-65	Ref		0.65	Ref		0.50
>= 65	0.84 (0.81-0.86)	<.001	0.61	0.81 (0.79-0.83)	<.001	0.44
**Race and Ethnicity**						
White	Ref		0.63	Ref		0.45
Asian	1.15 (1.10-1.20)	<.001	0.66	1.30 (1.25-1.36)	<.001	0.52
Black or African American	0.90 (0.84-0.95)	<.001	0.60	1.20 (1.13-1.28)	<.001	0.50
Native American or Alaska Native	1.31 (1.09-1.57)	0.004	0.69	1.13 (0.95-1.34)	0.17	0.48
Native Hawaiian or Other Pacific Islander	1.32 (1.11-1.57)	0.001	0.69	1.18 (1.00-1.38)	0.05	0.49
Unknown/Declined	0.73 (0.62-0.85)	<.001	0.55	0.70 (0.60-0.82)	<.001	0.37
Other	1.08 (1.04-1.14)	0.008	0.64	1.25 (1.20-1.31)	<.001	0.51
**Insurance**						
Private	Ref		0.65	Ref		0.48
Medicaid	0.98 (0.94-1.03)	0.47	0.65	1.16 (1.12-1.21)	<.001	0.52
Medicare	0.83 (0.80-0.86)	<.001	0.61	0.88 (0.85-0.92)	<.001	0.45
Other	1.29 (0.88-1.89)	0.19	0.71	0.57 (0.40-0.82)	0.003	0.35
Uninsured/Self-Pay	0.77 (0.61-0.99)	0.04	0.59	0.58 (0.45-0.75)	<.001	0.35
Unspecified	0.56 (0.49-0.63)	<.001	0.51	0.47 (0.41-0.54)	<.001	0.30
**Cancer Category**						
Prostate	Ref		0.51	Ref		0.22
Bladder and ureter	1.33 (1.20-1.48)	<.001	0.58	1.33 (1.20-1.48)	<.001	0.47
Bone soft tissue and sarcoma	1.85 (1.68-2.04)	<.001	0.66	4.75 (4.29-5.26)	<.001	0.57
Breast	1.24 (1.13-1.35)	<.001	0.56	2.61 (2.38-2.87)	<.001	0.42
CNS	1.62 (1.47-1.78)	<.001	0.63	2.36 (2.13-2.61)	<.001	0.40
Colorectal	1.81 (1.64-2.00)	<.001	0.65	3.46 (3.13-3.83)	<.001	0.49
GI other	1.80 (1.57-2.07)	<.001	0.65	3.27 (2.84-3.75)	<.001	0.48
Gynecologic	1.67 (1.52-1.83)	<.001	0.63	3.12 (2.83-3.44)	<.001	0.47
Head and neck	1.35 (1.20-1.53)	<.001	0.58	3.79 (3.33-4.31)	<.001	0.52
Hematologic	2.07 (1.94-2.21)	<.001	0.68	3.74 (3.47-4.03)	<.001	0.51
Kidney and renal pelvis	1.14 (1.00-1.30)	0.05	0.54	2.06 (1.80-2.37)	<.001	0.37
Liver and bile duct	1.41 (1.30-1.54)	<.001	0.60	2.50 (2.28-2.75)	<.001	0.41
Lung	1.42 (1.26-1.60)	<.001	0.60	3.11 (2.75-3.52)	<.001	0.47
Melanoma	1.02 (0.85-1.23)	0.80	0.52	2.43 (2.01-2.93)	<.001	0.41
Multiple Diagnoses	1.85 (1.63-2.11)	<.001	0.66	4.68 (4.11-5.33)	<.001	0.57
Non melanoma skin	0.81 (0.74-0.89)	<.001	0.46	1.59 (1.43-1.77)	<.001	0.31
Other	1.37 (1.25-1.50)	<.001	0.59	2.44 (2.21-2.69)	<.001	0.41
Pancreatic	1.91 (1.68-2.17)	<.001	0.67	3.72 (3.27-4.23)	<.001	0.51
Unspecified malignant neoplasm	2.42 (2.27-2.59)	<.001	0.72	5.07 (4.71-5.47)	<.001	0.59

© 2025 Chang C et al. *JAMA Network Open.*

eTable 4. Univariable Analysis of Fever, Weakness, Edema, Dyspnea, and Headache Documentation Across Patient Demographics

Variables	Fever			Generalized muscle weakness			Severity Edema			Dyspnea			Headache		
	OR	p-value	Predicted Margins	OR	OR	Predicted Margins	OR	p-value	Predicted Margins	OR	p-value	Predicted Margins	OR	p-value	Predicted Margins
**Sex**															
Male	Ref		0.41	Ref		0.40	Ref		0.38	Ref		0.37	Ref		0.32
Female	1.02 (0.99-1.05)	0.19	0.41	1.07 (1.04-1.10)	<.001	0.41	1.13 (1.09-1.16)	<.001	0.41	1.12 (1.09-1.15)	<.001	0.39	1.33 (1.29-1.38)	<.001	0.39
Unknown	1.02 (0.39-2.67)	0.97	0.41	0.83 (0.31-2.26)	0.72	0.35	0.10 (0.01-0.76)	0.03	0.06	0.72 (0.25-2.05)	0.54	0.29	0.88 (0.31-2.48)	0.80	0.29
**Age**															
18-65	Ref		0.45	Ref		0.40	Ref		0.40	Ref		0.37	Ref		0.40
>= 65	0.67 (0.65-0.70)	<.001	0.35	1.02 (0.99-1.06)	0.14	0.41	0.94 (0.92-0.97)	<.001	0.39	1.09 (1.05-1.12)	<.001	0.39	0.57 (0.55-0.59)	<.001	0.28
**Race and Ethnicity**															
White	Ref		0.38	Ref		0.37	Ref		0.38	Ref		0.36	Ref		0.34
Asian	1.42 (1.36-1.48)	<.001	0.46	1.40 (1.34-1.46)	<.001	0.45	1.19 (1.15-1.24)	<.001	0.43	1.36 (1.31-1.42)	<.001	0.43	1.07 (1.02-1.11)	0.002	0.36
Black or African American	1.03 (0.97-1.10)	0.28	0.38	1.36 (1.28-1.44)	<.001	0.45	1.21 (1.14-1.28)	<.001	0.43	1.26 (1.19-1.34)	<.001	0.41	1.03 (0.96-1.09)	0.41	0.35
Native American or Alaska Native	1.22 (1.02-1.45)	0.03	0.42	1.10 (0.92-1.31)	0.28	0.40	0.95 (0.80-1.14)	0.58	0.37	1.12 (0.94-1.34)	0.21	0.38	1.29 (1.09-1.54)	0.004	0.40
Native Hawaiian or Other Pacific Islander	1.23 (1.05-1.45)	0.01	0.43	1.31 (1.12-1.54)	0.001	0.44	1.37 (1.17-1.60)	<.001	0.46	1.30 (1.10-1.52)	0.001	0.42	1.04 (0.88-1.22)	0.68	0.35
Unknown/Declined	0.62 (0.52-0.74)	<.001	0.27	0.83 (0.71-0.98)	0.03	0.33	0.70 (0.60-0.83)	<.001	0.30	0.85 (0.72-1.01)	0.06	0.32	0.86 (0.73-1.01)	0.07	0.31
Other	1.56 (1.50-1.63)	<.001	0.49	1.28 (1.23-1.34)	<.001	0.43	1.10 (1.06-1.15)	<.001	0.41	1.13 (1.08-1.18)	<.001	0.38	1.25 (1.20-1.31)	<.001	0.39
**Insurance**															
Private	Ref		0.42	Ref		0.39	Ref		0.40	Ref		0.36	Ref		0.38
Medicaid	1.34 (1.29-1.40)	<.001	0.49	1.26 (1.21-1.31)	<.001	0.44	1.11 (1.07-1.16)	<.001	0.42	1.16 (1.11-1.21)	<.001	0.40	1.10 (1.06-1.15)	<.001	0.40
Medicare	0.76 (0.73-0.79)	<.001	0.35	1.11 (1.07-1.15)	<.001	0.41	0.99 (0.95-1.02)	0.46	0.39	1.14 (1.10-1.18)	<.001	0.39	0.66 (0.63-0.68)	<.001	0.29
Other	1.20 (0.85-1.71)	0.30	0.46	1.47 (1.04-2.09)	0.03	0.48	0.73 (0.50-1.06)	0.09	0.32	1.21 (0.85-1.73)	0.28	0.41	1.20 (0.85-1.71)	0.30	0.43
Uninsured/Self-Pay	0.75 (0.58-0.96)	0.02	0.35	0.53 (0.40-0.70)	<.001	0.25	0.67 (0.52-0.87)	0.003	0.31	0.73 (0.56-0.95)	0.02	0.29	0.59 (0.45-0.77)	<.001	0.26
Unspecified	0.42 (0.36-0.49)	<.001	0.23	0.51 (0.44-0.59)	<.001	0.24	0.49 (0.43-0.57)	<.001	0.24	0.58 (0.50-0.67)	<.001	0.25	0.54 (0.46-0.62)	<.001	0.25
**Cancer Category**															
Prostate	Ref		0.18	Ref		0.15	Ref		0.18	Ref		0.18	Ref		0.18
Bladder and ureter	2.84 (2.53-3.18)	<.001	0.38	2.33 (2.06-2.63)	<.001	0.29	1.95 (1.73-2.19)	<.001	0.30	2.07 (1.85-2.33)	<.001	0.32	1.29 (1.14-1.47)	<.001	0.22
Bone soft tissue and sarcoma	3.92 (3.53-4.36)	<.001	0.46	3.76 (3.37-4.20)	<.001	0.40	3.69 (3.32-4.10)	<.001	0.45	1.96 (1.76-2.19)	<.001	0.31	3.79 (3.41-4.22)	<.001	0.45
Breast	2.18 (1.97-2.41)	<.001	0.32	2.19 (1.97-2.43)	<.001	0.28	2.42 (2.19-2.68)	<.001	0.35	1.71 (1.54-1.89)	<.001	0.28	2.39 (2.16-2.64)	<.001	0.34
CNS	1.30 (1.15-1.45)	<.001	0.22	5.95 (5.34-6.63)	<.001	0.52	3.25 (2.93-3.61)	<.001	0.42	1.38 (1.23-1.55)	<.001	0.24	9.35 (8.40-10.41)	<.001	0.67
Colorectal	2.78 (2.50-3.10)	<.001	0.38	3.23 (2.89-3.61)	<.001	0.37	1.95 (1.75-2.18)	<.001	0.30	2.52 (2.27-2.80)	<.001	0.36	1.80 (1.61-2.01)	<.001	0.28
GI other	2.15 (1.85-2.49)	<.001	0.32	3.81 (3.30-4.41)	<.001	0.41	1.97 (1.69-2.29)	<.001	0.30	2.94 (2.55-3.39)	<.001	0.40	1.72 (1.48-2.01)	<.001	0.27
Gynecologic	2.12 (1.91-2.36)	<.001	0.32	2.77 (2.49-3.09)	<.001	0.33	1.81 (1.62-2.01)	<.001	0.28	2.02 (1.81-2.24)	<.001	0.31	2.16 (1.94-2.40)	<.001	0.31
Head and neck	1.55 (1.34-1.79)	<.001	0.25	3.13 (2.73-3.59)	<.001	0.36	3.38 (2.96-3.85)	<.001	0.43	2.91 (2.54-3.32)	<.001	0.40	1.98 (1.72-2.28)	<.001	0.30
Hematologic	6.76 (6.24-7.32)	<.001	0.59	5.99 (5.51-6.51)	<.001	0.52	4.27 (3.94-4.62)	<.001	0.48	3.87 (3.58-4.18)	<.001	0.47	3.18 (2.94-3.45)	<.001	0.40
Kidney and renal pelvis	1.70 (1.46-1.97)	<.001	0.27	2.70 (2.34-3.13)	<.001	0.33	1.98 (1.71-2.29)	<.001	0.30	2.04 (1.77-2.36)	<.001	0.32	1.76 (1.52-2.05)	<.001	0.27
Liver and bile duct	3.29 (2.98-3.63)	<.001	0.42	3.47 (3.13-3.85)	<.001	0.38	3.48 (3.16-3.84)	<.001	0.43	2.72 (2.46-3.00)	<.001	0.38	2.18 (1.97-2.41)	<.001	0.32
Lung	2.82 (2.48-3.20)	<.001	0.38	3.75 (3.29-4.26)	<.001	0.40	2.48 (2.18-2.82)	<.001	0.35	5.00 (4.41-5.67)	<.001	0.53	2.12 (1.86-2.42)	<.001	0.31
Melanoma	1.98 (1.62-2.42)	<.001	0.30	3.17 (2.61-3.85)	<.001	0.36	2.98 (2.46-3.60)	<.001	0.40	1.78 (1.45-2.19)	<.001	0.29	3.05 (2.52-3.69)	<.001	0.39
Multiple Diagnoses	4.48 (3.92-5.11)	<.001	0.49	4.93 (4.31-5.65)	<.001	0.47	4.83 (4.23-5.51)	<.001	0.51	4.18 (3.67-4.77)	<.001	0.49	2.71 (2.37-3.11)	<.001	0.37
Non melanoma skin	1.70 (1.53-1.91)	<.001	0.27	2.86 (2.56-3.20)	<.001	0.34	3.11 (2.80-3.46)	<.001	0.41	2.53 (2.27-2.81)	<.001	0.36	1.65 (1.47-1.84)	<.001	0.26
Other	2.33 (2.10-2.58)	<.001	0.34	2.11 (1.89-2.35)	<.001	0.27	1.95 (1.76-2.16)	<.001	0.30	2.21 (1.99-2.44)	<.001	0.33	2.25 (2.03-2.50)	<.001	0.32
Pancreatic	3.74 (3.28-4.28)	<.001	0.45	3.27 (2.85-3.76)	<.001	0.37	2.60 (2.27-2.98)	<.001	0.36	2.45 (2.14-2.80)	<.001	0.36	1.16 (1.00-1.36)	0.06	0.20
Unspecified malignant neoplasm	3.65 (3.37-3.95)	<.001	0.44	4.76 (4.38-5.18)	<.001	0.46	3.77 (3.48-4.08)	<.001	0.45	3.40 (3.14-3.68)	<.001	0.43	3.02 (2.79-3.28)	<.001	0.39

© 2025 Chang C et al. *JAMA Network Open.*

eTable 5. Multivariable Analysis of Fatigue and Constipation Documentation Across Patient Demographics

Variables	Fatigue		Constipation	
	OR	p-value	OR	p-value
**Sex**				
Male	Ref		Ref	
Female	1.08 (1.05-1.12)	<.001	1.13 (1.09-1.16)	<.001
Unknown	1.21 (0.42-3.48)	0.73	0.69 (0.26-1.85)	0.46
**Age**				
18-65	Ref		Ref	
>= 65	0.95 (0.91-1.00)	0.03	0.92 (0.89-0.96)	<.001
**Race and Ethnicity**				
White	Ref		Ref	
Asian	1.05 (1.01-1.10)	0.02	1.17 (1.13-1.22)	<.001
Black or African American	0.87 (0.81-0.92)	<.001	1.14 (1.07-1.21)	<.001
Native American or Alaska Native	1.25 (1.03-1.50)	0.02	1.04 (0.87-1.23)	0.68
Native Hawaiian or Other Pacific Islander	1.22 (1.03-1.45)	0.03	1.06 (0.90-1.25)	0.46
Unknown/Declined	0.73 (0.62-0.85)	<.001	0.71 (0.61-0.84)	<.001
Other	0.97 (0.92-1.01)	0.13	1.12 (1.07-1.17)	<.001
**Insurance**				
Private	Ref		Ref	
Medicaid	0.93 (0.89-0.97)	0.001	1.03 (0.99-1.08)	0.12
Medicare	0.90 (0.86-0.94)	<.001	0.97 (0.93-1.02)	0.20
Other	1.27 (0.86-1.86)	0.23	0.54 (0.38-0.79)	0.001
Uninsured/Self-Pay	0.85 (0.66-1.09)	0.19	0.67 (0.52-0.87)	0.002
Unspecified	0.70 (0.61-0.80)	<.001	0.67 (0.58-0.78)	<.001
**Cancer Category**				
Prostate	Ref		Ref	
Bladder and ureter	1.30 (1.18-1.45)	<.001	3.05 (2.73-3.40)	<.001
Bone soft tissue and sarcoma	1.72 (1.56-1.90)	<.001	4.12 (3.71-4.57)	<.001
Breast	1.08 (0.98-1.18)	0.12	2.17 (1.96-2.40)	<.001
CNS	1.47 (1.33-1.62)	<.001	2.11 (1.90-2.34)	<.001
Colorectal	1.70 (1.54-1.87)	<.001	3.07 (2.77-3.40)	<.001
GI other	1.67 (1.45-1.93)	<.001	2.92 (2.54-3.36)	<.001
Gynecologic	1.48 (1.34-1.64)	<.001	2.55 (2.30-2.84)	<.001
Head and neck	1.29 (1.14-1.46)	<.001	3.43 (3.02-3.91)	<.001
Hematologic	1.91 (1.79-2.05)	<.001	3.27 (3.03-3.54)	<.001
Kidney and renal pelvis	1.07 (0.94-1.22)	0.30	1.84 (1.60-2.11)	<.001
Liver and bile duct	1.32 (1.21-1.44)	<.001	2.19 (1.99-2.41)	<.001
Lung	1.35 (1.20-1.52)	<.001	2.77 (2.44-3.13)	<.001
Melanoma	0.94 (0.79-1.13)	0.54	2.26 (1.87-2.74)	<.001
Multiple Diagnoses	1.75 (1.53-1.99)	<.001	4.17 (3.65-4.75)	<.001
Non melanoma skin	0.77 (0.70-0.84)	<.001	1.50 (1.35-1.67)	<.001
Other	1.24 (1.13-1.36)	<.001	2.12 (1.92-2.34)	<.001
Pancreatic	1.77 (1.56-2.02)	<.001	3.31 (2.91-3.78)	<.001
Unspecified malignant neoplasm	2.21 (2.06-2.37)	<.001	4.42 (4.09-4.78)	<.001

© 2025 Chang C et al. *JAMA Network Open.*

eTable 6. Multivariable Analysis of Fever, Weakness, Edema, Dyspnea, and Headache Documentation Across Patient Demographics

Variables	Fever		Generalized muscle weakness		Severity Edema		Dyspnea		Headache	
	OR	p-value	OR	p-value	OR	p-value	OR	p-value	OR	p-value
**Sex**										
Male	Ref		Ref		Ref		Ref		Ref	
Female	0.98 (0.95-1.01)	0.24	1.02 (0.99-1.06)	0.23	1.09 (1.06-1.13)	<.001	1.09 (1.05-1.12)	<.001	1.29 (1.25-1.34)	<.001
Unknown	0.81 (0.30-2.17)	0.67	0.75 (0.28-2.06)	0.58	0.09 (0.01-0.66)	0.02	0.63 (0.22-1.80)	0.39	0.80 (0.28-2.30)	0.68
**Age**										
18-65	Ref		Ref		Ref					
>= 65	0.81 (0.78-0.85)	<.001	1.18 (1.13-1.24)	<.001	1.05 (1.00-1.10)	0.03	1.13 (1.08-1.18)	<.001	0.69 (0.66-0.72)	<.001
**Race and Ethnicity**										
White	Ref		Ref		Ref					
Asian	1.30 (1.25-1.36)	<.001	1.32 (1.26-1.37)	<.001	1.13 (1.08-1.18)	<.001	1.25 (1.20-1.30)	<.001	1.04 (1.00-1.09)	0.08
Black or African American	0.98 (0.92-1.05)	0.61	1.42 (1.33-1.51)	<.001	1.24 (1.17-1.32)	<.001	1.27 (1.19-1.35)	<.001	1.01 (0.94-1.08)	0.81
Native American or Alaska Native	1.16 (0.98-1.39)	0.09	1.19 (1.00-1.43)	0.05	0.98 (0.82-1.17)	0.80	1.12 (0.94-1.34)	0.20	1.25 (1.04-1.49)	0.02
Native Hawaiian or Other Pacific Islander	1.15 (0.97-1.35)	0.11	1.34 (1.14-1.57)	<.001	1.40 (1.19-1.64)	<.001	1.25 (1.06-1.47)	0.007	1.00 (0.85-1.19)	0.96
Unknown/Declined	0.63 (0.53-0.75)	<.001	0.90 (0.76-1.07)	0.22	0.74 (0.63-0.88)	0.001	0.90 (0.76-1.06)	0.20	0.83 (0.70-0.99)	0.04
Other	1.20 (1.15-1.26)	<.001	1.15 (1.10-1.20)	<.001	0.99 (0.95-1.04)	0.71	1.03 (0.98-1.08)	0.23	1.08 (1.03-1.13)	0.001
**Insurance**										
Private	Ref		Ref		Ref					
Medicaid	1.12 (1.07-1.17)	<.001	1.14 (1.10-1.19)	<.001	1.05 (1.00-1.09)	0.03	1.08 (1.03-1.13)	0.001	1.01 (0.96-1.05)	0.77
Medicare	0.88 (0.84-0.92)	<.001	1.07 (1.02-1.12)	0.003	1.00 (0.96-1.05)	0.91	1.06 (1.02-1.11)	0.01	0.92 (0.88-0.97)	0.001
Other	1.29 (0.90-1.85)	0.17	1.44 (1.01-2.06)	0.05	0.70 (0.48-1.02)	0.06	1.25 (0.87-1.79)	0.23	1.14 (0.79-1.64)	0.49
Uninsured/Self-Pay	0.96 (0.74-1.25)	0.78	0.58 (0.43-0.77)	<.001	0.78 (0.60-1.02)	0.07	0.91 (0.69-1.19)	0.48	0.54 (0.41-0.72)	<.001
Unspecified	0.61 (0.53-0.72)	<.001	0.77 (0.66-0.90)	0.001	0.73 (0.63-0.85)	<.001	0.78 (0.67-0.91)	0.001	0.78 (0.67-0.90)	0.001
**Cancer Category**										
Prostate	Ref		Ref		Ref		Ref		Ref	
Bladder and ureter	2.84 (2.53-3.19)	<.001	2.23 (1.98-2.52)	<.001	1.87 (1.66-2.10)	<.001	1.97 (1.75-2.21)	<.001	1.23 (1.08-1.40)	0.002
Bone soft tissue and sarcoma	3.27 (2.93-3.64)	<.001	3.71 (3.32-4.15)	<.001	3.48 (3.12-3.87)	<.001	1.89 (1.69-2.12)	<.001	2.84 (2.55-3.17)	<.001
Breast	1.89 (1.70-2.10)	<.001	2.20 (1.97-2.46)	<.001	2.20 (1.98-2.45)	<.001	1.60 (1.43-1.78)	<.001	1.58 (1.42-1.76)	<.001
CNS	1.10 (0.98-1.24)	0.10	6.39 (5.73-7.13)	<.001	3.18 (2.85-3.53)	<.001	1.40 (1.25-1.57)	<.001	7.14 (6.40-7.97)	<.001
Colorectal	2.43 (2.18-2.71)	<.001	3.18 (2.84-3.56)	<.001	1.84 (1.64-2.05)	<.001	2.42 (2.17-2.70)	<.001	1.40 (1.25-1.57)	<.001
GI other	1.89 (1.62-2.20)	<.001	3.59 (3.10-4.17)	<.001	1.84 (1.58-2.14)	<.001	2.73 (2.36-3.16)	<.001	1.44 (1.23-1.69)	<.001
Gynecologic	1.80 (1.61-2.01)	<.001	2.68 (2.39-3.01)	<.001	1.62 (1.44-1.81)	<.001	1.85 (1.66-2.07)	<.001	1.42 (1.27-1.59)	<.001
Head and neck	1.40 (1.21-1.62)	<.001	2.95 (2.57-3.39)	<.001	3.17 (2.77-3.62)	<.001	2.71 (2.37-3.10)	<.001	1.69 (1.46-1.94)	<.001
Hematologic	5.72 (5.27-6.22)	<.001	5.91 (5.42-6.45)	<.001	4.06 (3.74-4.41)	<.001	3.76 (3.46-4.08)	<.001	2.45 (2.26-2.67)	<.001
Kidney and renal pelvis	1.52 (1.31-1.76)	<.001	2.58 (2.23-2.99)	<.001	1.85 (1.60-2.14)	<.001	1.92 (1.66-2.22)	<.001	1.45 (1.25-1.68)	<.001
Liver and bile duct	2.77 (2.50-3.07)	<.001	3.28 (2.95-3.64)	<.001	3.29 (2.97-3.64)	<.001	2.57 (2.32-2.84)	<.001	1.76 (1.59-1.96)	<.001
Lung	2.65 (2.32-3.02)	<.001	3.36 (2.95-3.84)	<.001	2.24 (1.97-2.56)	<.001	4.45 (3.92-5.05)	<.001	1.83 (1.60-2.10)	<.001
Melanoma	1.89 (1.54-2.32)	<.001	3.41 (2.80-4.15)	<.001	2.92 (2.41-3.54)	<.001	1.81 (1.47-2.22)	<.001	2.46 (2.02-2.98)	<.001
Multiple Diagnoses	4.07 (3.56-4.66)	<.001	4.70 (4.10-5.39)	<.001	4.49 (3.92-5.12)	<.001	3.94 (3.45-4.50)	<.001	2.23 (1.94-2.56)	<.001
Non melanoma skin	1.72 (1.53-1.93)	<.001	2.92 (2.61-3.27)	<.001	3.01 (2.70-3.35)	<.001	2.47 (2.22-2.75)	<.001	1.45 (1.30-1.63)	<.001
Other	1.98 (1.78-2.20)	<.001	2.14 (1.92-2.40)	<.001	1.86 (1.67-2.06)	<.001	2.16 (1.95-2.40)	<.001	1.65 (1.48-1.83)	<.001
Pancreatic	3.42 (2.99-3.91)	<.001	3.12 (2.72-3.59)	<.001	2.42 (2.11-2.78)	<.001	2.28 (1.98-2.61)	<.001	0.95 (0.81-1.11)	0.53
Unspecified malignant neoplasm	3.14 (2.90-3.41)	<.001	4.69 (4.30-5.11)	<.001	3.53 (3.25-3.83)	<.001	3.24 (2.99-3.52)	<.001	2.30 (2.12-2.50)	<.001

© 2025 Chang C et al. *JAMA Network Open.*

eTable 7: GEE analysis for symptom burden across demographic and cancer characteristics

Symptom Burden (>10 Symptoms)

Variables	Univariable			Multivariable	
	OR	p-value	Predicted Margins	OR	p-value
**Sex**					
Male	Ref		0.38	Ref	
Female	1.28 (1.23-1.33)	<.001	0.44	1.12 (1.07-1.17)	<.001
Unknown	0.65 (0.19-2.21)	0.49	0.29	0.54 (0.16-1.80)	0.32
**Age**					
18-65	Ref		0.42	Ref	
>= 65	0.93 (0.89-0.96)	<.001	0.40	1.01 (0.96-1.07)	0.66
**Race and Ethnicity**					
White	Ref		0.39	Ref	
Asian	1.33 (1.26-1.40)	<.001	0.46	1.17 (1.11-1.24)	<.001
Black or African American	1.30 (1.20-1.41)	<.001	0.45	1.23 (1.13-1.34)	<.001
Native American or Alaska Native	1.35 (1.08-1.68)	0.009	0.46	1.25 (1.00-1.57)	0.05
Native Hawaiian or Other Pacific Islander	1.27 (1.02-1.58)	0.03	0.45	1.16 (0.93-1.45)	0.19
Unknown/Declined	0.67 (0.55-0.81)	<.001	0.30	0.68 (0.56-0.82)	<.001
Other	1.18 (1.12-1.25)	<.001	0.43	1.06 (1.00-1.12)	0.06
**Insurance**					
Private	Ref		0.41	Ref	
Medicaid	1.19 (1.12-1.25)	<.001	0.45	1.05 (0.99-1.11)	0.10
Medicare	0.99 (0.94-1.03)	0.54	0.40	0.99 (0.93-1.05)	0.69
Other	0.78 (0.50-1.23)	0.29	0.35	0.77 (0.50-1.20)	0.25
Uninsured/Self-Pay	0.61 (0.47-0.81)	<.001	0.30	0.70 (0.53-0.93)	0.01
Unspecified	0.45 (0.38-0.53)	<.001	0.24	0.61 (0.52-0.73)	<.001
**Cancer Category**					
Prostate	Ref		0.18	Ref	
Bladder and ureter	2.91 (2.55-3.32)	<.001	0.38	2.76 (2.42-3.16)	<.001
Bone soft tissue and sarcoma	3.47 (3.03-3.98)	<.001	0.43	3.13 (2.73-3.60)	<.001
Breast	2.63 (2.35-2.95)	<.001	0.36	2.25 (2.00-2.55)	<.001
CNS	2.34 (2.07-2.65)	<.001	0.33	2.18 (1.92-2.47)	<.001
Colorectal	4.03 (3.56-4.58)	<.001	0.46	3.69 (3.25-4.20)	<.001
GI other	3.34 (2.82-3.97)	<.001	0.42	3.02 (2.54-3.60)	<.001
Gynecologic	2.78 (2.46-3.14)	<.001	0.37	2.34 (2.06-2.67)	<.001
Head and neck	4.23 (3.63-4.93)	<.001	0.47	3.84 (3.30-4.48)	<.001
Hematologic	5.26 (4.80-5.78)	<.001	0.53	4.78 (4.35-5.26)	<.001
Kidney and renal pelvis	1.78 (1.49-2.13)	<.001	0.28	1.62 (1.35-1.93)	<.001
Liver and bile duct	2.97 (2.63-3.36)	<.001	0.39	2.66 (2.35-3.01)	<.001
Lung	3.57 (3.06-4.16)	<.001	0.43	3.17 (2.71-3.70)	<.001
Melanoma	2.24 (1.79-2.80)	<.001	0.32	2.13 (1.70-2.67)	<.001
Multiple Diagnoses	5.58 (4.65-6.69)	<.001	0.54	5.05 (4.21-6.06)	<.001
Non melanoma skin	2.09 (1.84-2.37)	<.001	0.31	1.96 (1.72-2.23)	<.001
Other	2.26 (2.01-2.54)	<.001	0.32	2.03 (1.80-2.30)	<.001
Pancreatic	4.39 (3.74-5.15)	<.001	0.48	3.94 (3.35-4.63)	<.001
Unspecified malignant neoplasm	5.33 (4.88-5.83)	<.001	0.53	4.75 (4.33-5.22)	<.001

© 2025 Chang C et al. *JAMA Network Open.*

eTable 8: Univariable GEE analysis for documented symptoms across demographic and cancer characteristics

Variables	Pain			Nausea			Vomiting			Fatigue			Constipation		
	OR	p-value	Predicted Margins	OR	OR	Predicted Margins	OR	p-value	Predicted Margins	OR	p-value	Predicted Margins	OR	p-value	Predicted Margins
**Sex**															
Male	Ref		0.91	Ref		0.80	Ref		0.70	Ref		0.60	Ref		0.36
Female	1.18 (1.11-1.26)	<.001	0.92	1.20 (1.14-1.26)	<.001	0.83	1.12 (1.07-1.17)	<.001	0.73	1.13 (1.08-1.17)	<.001	0.63	1.36 (1.31-1.42)	<.001	0.44
Unknown	0.68 (0.17-2.78)	0.59	0.87	0.53 (0.17-1.68)	0.28	0.68	0.95 (0.30-3.02)	0.93	0.69	1.15 (0.31-4.21)	0.84	0.63	1.08 (0.33-3.52)	0.89	0.38
**Age**															
18-65	Ref		0.92	Ref		0.84	Ref		0.74	Ref		0.62	Ref		0.41
>= 65	0.72 (0.67-0.77)	<.001	0.90	0.67 (0.63-0.70)	<.001	0.78	0.72 (0.69-0.75)	<.001	0.67	0.91 (0.88-0.95)	<.001	0.60	0.87 (0.83-0.90)	<.001	0.38
**Race and Ethnicity**															
White	Ref		0.91	Ref		0.82	Ref		0.72	Ref		0.61	Ref		0.38
Asian	0.82 (0.76-0.89)	<.001	0.90	0.88 (0.82-0.95)	<.001	0.80	0.84 (0.80-0.90)	<.001	0.69	1.15 (1.09-1.21)	<.001	0.64	1.34 (1.27-1.42)	<.001	0.45
Black or African American	0.96 (0.83-1.09)	0.51	0.91	0.70 (0.63-0.77)	<.001	0.76	0.69 (0.64-0.75)	<.001	0.65	0.88 (0.81-0.95)	0.002	0.58	1.24 (1.14-1.35)	<.001	0.43
Native American or Alaska Native	1.27 (0.88-1.84)	0.21	0.93	0.92 (0.70-1.20)	0.53	0.81	0.92 (0.73-1.16)	0.50	0.71	1.34 (1.07-1.68)	0.01	0.68	1.15 (0.91-1.44)	0.24	0.41
Native Hawaiian or Other Pacific Islander	1.17 (0.83-1.65)	0.36	0.93	1.00 (0.75-1.34)	0.99	0.82	0.89 (0.70-1.12)	0.32	0.70	1.25 (1.00-1.56)	0.05	0.66	1.35 (1.08-1.68)	0.009	0.45
Unknown/Declined	1.03 (0.77-1.37)	0.86	0.92	0.88 (0.71-1.09)	0.25	0.80	1.04 (0.86-1.26)	0.71	0.73	0.71 (0.60-0.85)	<.001	0.53	0.79 (0.65-0.95)	0.01	0.33
Other	1.12 (1.01-1.23)	0.03	0.92	1.04 (0.97-1.12)	0.27	0.83	0.99 (0.93-1.05)	0.77	0.72	0.99 (0.93-1.05)	0.90	0.61	1.18 (1.11-1.25)	<.001	0.42
**Insurance**															
Private	Ref		0.92	Ref		0.84	Ref		0.74	Ref		0.63	Ref		0.40
Medicaid	1.21 (1.10-1.33)	<.001	0.93	0.94 (0.87-1.01)	0.08	0.83	0.98 (0.92-1.04)	0.56	0.73	0.95 (0.90-1.00)	0.05	0.61	1.16 (1.09-1.23)	<.001	0.44
Medicare	0.79 (0.74-0.85)	<.001	0.90	0.67 (0.63-0.70)	<.001	0.78	0.73 (0.69-0.76)	<.001	0.67	0.89 (0.85-0.93)	<.001	0.60	0.94 (0.90-0.99)	0.01	0.39
Other	1.12 (0.51-2.48)	0.78	0.92	0.83 (0.49-1.41)	0.49	0.81	1.22 (0.72-2.05)	0.46	0.77	1.10 (0.70-1.73)	0.66	0.65	0.59 (0.37-0.95)	0.03	0.29
Uninsured/Self-Pay	0.76 (0.51-1.13)	0.18	0.89	0.89 (0.62-1.27)	0.52	0.82	1.11 (0.80-1.55)	0.52	0.76	0.85 (0.65-1.11)	0.24	0.59	0.71 (0.53-0.95)	0.02	0.32
Unspecified	0.88 (0.69-1.11)	0.28	0.91	0.64 (0.54-0.75)	<.001	0.77	0.92 (0.79-1.08)	0.31	0.72	0.60 (0.52-0.69)	<.001	0.50	0.53 (0.45-0.62)	<.001	0.26
**Cancer Category**															
Prostate	Ref		0.95	Ref		0.86	Ref		0.83	Ref		0.51	Ref		0.18
Bladder and ureter	0.83 (0.65-1.06)	0.13	0.94	0.66 (0.56-0.77)	<.001	0.81	0.60 (0.52-0.70)	<.001	0.75	1.32 (1.17-1.50)	<.001	0.58	1.32 (1.17-1.50)	<.001	0.42
Bone soft tissue and sarcoma	1.27 (0.95-1.69)	0.11	0.96	0.84 (0.71-1.01)	0.06	0.84	0.70 (0.60-0.82)	<.001	0.78	1.57 (1.39-1.77)	<.001	0.62	3.41 (2.95-3.94)	<.001	0.42
Breast	0.63 (0.51-0.76)	<.001	0.93	0.69 (0.60-0.79)	<.001	0.81	0.58 (0.51-0.66)	<.001	0.74	1.24 (1.12-1.37)	<.001	0.56	3.22 (2.87-3.60)	<.001	0.41
CNS	0.26 (0.22-0.32)	<.001	0.84	0.60 (0.52-0.70)	<.001	0.79	0.43 (0.38-0.49)	<.001	0.69	1.53 (1.37-1.71)	<.001	0.61	2.43 (2.14-2.75)	<.001	0.34
Colorectal	0.73 (0.57-0.92)	0.009	0.94	0.98 (0.82-1.18)	0.87	0.86	0.73 (0.63-0.85)	<.001	0.79	1.69 (1.50-1.90)	<.001	0.64	4.51 (3.96-5.13)	<.001	0.49
GI other	0.68 (0.49-0.94)	0.02	0.93	0.85 (0.68-1.07)	0.17	0.84	0.77 (0.63-0.94)	0.010	0.79	1.67 (1.41-1.97)	<.001	0.64	3.23 (2.69-3.88)	<.001	0.41
Gynecologic	1.15 (0.90-1.47)	0.25	0.96	1.11 (0.95-1.31)	0.19	0.88	0.93 (0.81-1.08)	0.35	0.82	1.48 (1.33-1.65)	<.001	0.61	3.01 (2.66-3.42)	<.001	0.39
Head and neck	0.89 (0.66-1.21)	0.47	0.95	0.92 (0.75-1.12)	0.40	0.85	0.68 (0.57-0.81)	<.001	0.77	1.33 (1.16-1.54)	<.001	0.58	4.27 (3.65-5.01)	<.001	0.48
Hematologic	0.29 (0.25-0.33)	<.001	0.85	0.59 (0.53-0.66)	<.001	0.79	0.30 (0.27-0.33)	<.001	0.60	1.78 (1.65-1.93)	<.001	0.65	3.66 (3.32-4.03)	<.001	0.44
Kidney and renal pelvis	0.68 (0.51-0.90)	0.008	0.93	0.60 (0.49-0.73)	<.001	0.79	0.50 (0.42-0.60)	<.001	0.72	1.05 (0.90-1.23)	0.51	0.52	1.63 (1.35-1.97)	<.001	0.26
Liver and bile duct	0.30 (0.25-0.36)	<.001	0.86	0.40 (0.35-0.46)	<.001	0.72	0.28 (0.25-0.32)	<.001	0.59	1.31 (1.17-1.47)	<.001	0.58	2.55 (2.25-2.89)	<.001	0.35
Lung	0.48 (0.38-0.62)	<.001	0.91	0.50 (0.42-0.60)	<.001	0.76	0.40 (0.34-0.48)	<.001	0.67	1.37 (1.19-1.59)	<.001	0.59	2.55 (2.15-3.02)	<.001	0.35
Melanoma	0.64 (0.43-0.94)	0.02	0.93	0.51 (0.39-0.66)	<.001	0.76	0.45 (0.35-0.56)	<.001	0.69	0.90 (0.72-1.12)	0.35	0.48	2.15 (1.69-2.74)	<.001	0.31
Multiple Diagnoses	0.54 (0.39-0.74)	<.001	0.91	0.65 (0.49-0.85)	0.002	0.80	0.47 (0.38-0.59)	<.001	0.70	1.72 (1.41-2.09)	<.001	0.64	4.99 (4.04-6.16)	<.001	0.52
Non melanoma skin	0.28 (0.23-0.34)	<.001	0.85	0.24 (0.21-0.27)	<.001	0.60	0.20 (0.18-0.23)	<.001	0.51	0.77 (0.68-0.86)	<.001	0.44	1.58 (1.37-1.81)	<.001	0.25
Other	0.84 (0.67-1.03)	0.10	0.94	0.86 (0.74-0.99)	0.04	0.84	0.69 (0.61-0.78)	<.001	0.78	1.32 (1.19-1.46)	<.001	0.58	2.66 (2.36-2.99)	<.001	0.36
Pancreatic	1.03 (0.74-1.42)	0.88	0.95	1.33 (1.04-1.70)	0.02	0.89	0.94 (0.77-1.15)	0.56	0.83	1.88 (1.60-2.20)	<.001	0.66	3.84 (3.24-4.55)	<.001	0.45
Unspecified malignant neoplasm	0.72 (0.61-0.84)	<.001	0.93	0.84 (0.75-0.93)	<.001	0.84	0.52 (0.47-0.58)	<.001	0.72	2.20 (2.04-2.37)	<.001	0.70	5.17 (4.72-5.68)	<.001	0.52

© 2025 Chang C et al. *JAMA Network Open.*

Variables	Fever			Generalized muscle weakness			Severity Edema			Dyspnea			Headache		
	OR	p-value	Predicted Margins	OR	p-value	Predicted Margins	OR	p-value	Predicted Margins	OR	p-value	Predicted Margins	OR	p-value	Predicted Margins
**Sex**															
Male	Ref		0.31	Ref		0.31	Ref		0.31	Ref		0.31	Ref		0.25
Female	1.11 (1.06-1.15)	<.001	0.34	1.10 (1.06-1.15)	<.001	0.33	1.19 (1.14-1.24)	<.001	0.35	1.14 (1.09-1.18)	<.001	0.33	1.49 (1.42-1.55)	<.001	0.34
Unknown	1.23 (0.41-3.71)	0.71	0.36	0.94 (0.26-3.36)	0.92	0.30	0.15 (0.02-1.07)	0.06	0.07	0.72 (0.24-2.16)	0.56	0.24	0.71 (0.17-2.96)	0.63	0.19
**Age**															
18-65	Ref		0.35	Ref		0.32	Ref		0.34	Ref		0.31	Ref		0.33
>= 65	0.79 (0.75-0.82)	<.001	0.30	1.08 (1.03-1.13)	<.001	0.33	0.95 (0.91-1.00)	0.03	0.33	1.10 (1.05-1.15)	<.001	0.33	0.65 (0.62-0.68)	<.001	0.24
**Race and Ethnicity**															
White	Ref		0.30	Ref		0.30	Ref		0.32	Ref		0.30	Ref		0.29
Asian	1.40 (1.32-1.49)	<.001	0.38	1.40 (1.32-1.48)	<.001	0.38	1.22 (1.15-1.29)	<.001	0.37	1.38 (1.30-1.46)	<.001	0.37	1.05 (0.98-1.11)	0.14	0.30
Black or African American	1.09 (1.00-1.19)	0.05	0.32	1.37 (1.26-1.50)	<.001	0.37	1.27 (1.16-1.38)	<.001	0.37	1.34 (1.22-1.46)	<.001	0.36	1.02 (0.93-1.12)	0.69	0.29
Native American or Alaska Native	1.20 (0.95-1.53)	0.13	0.35	1.14 (0.91-1.44)	0.26	0.33	0.98 (0.78-1.24)	0.88	0.32	1.14 (0.91-1.44)	0.25	0.33	1.35 (1.07-1.69)	0.010	0.35
Native Hawaiian or Other Pacific Islander	1.37 (1.09-1.71)	0.006	0.37	1.25 (1.00-1.57)	0.05	0.35	1.36 (1.09-1.71)	0.008	0.39	1.28 (1.02-1.60)	0.03	0.35	1.17 (0.91-1.49)	0.22	0.32
Unknown/Declined	0.67 (0.54-0.83)	<.001	0.23	0.89 (0.74-1.09)	0.26	0.28	0.73 (0.59-0.89)	0.002	0.26	0.93 (0.76-1.13)	0.45	0.28	0.93 (0.76-1.13)	0.44	0.27
Other	1.33 (1.25-1.42)	<.001	0.37	1.24 (1.16-1.31)	<.001	0.35	1.08 (1.02-1.15)	0.01	0.34	1.14 (1.07-1.21)	<.001	0.33	1.13 (1.06-1.20)	<.001	0.31
**Insurance**															
Private	Ref		0.34	Ref		0.31	Ref		0.33	Ref		0.31	Ref		0.32
Medicaid	1.18 (1.11-1.25)	<.001	0.38	1.24 (1.17-1.32)	<.001	0.36	1.11 (1.05-1.18)	<.001	0.36	1.16 (1.10-1.23)	<.001	0.34	1.06 (1.00-1.13)	0.04	0.34
Medicare	0.83 (0.79-0.87)	<.001	0.30	1.17 (1.12-1.23)	<.001	0.34	1.00 (0.96-1.05)	0.90	0.33	1.14 (1.09-1.20)	<.001	0.33	0.68 (0.65-0.72)	<.001	0.24
Other	1.08 (0.69-1.71)	0.73	0.36	1.14 (0.71-1.82)	0.59	0.33	0.68 (0.43-1.07)	0.09	0.25	1.15 (0.74-1.78)	0.55	0.34	1.11 (0.71-1.75)	0.65	0.35
Uninsured/Self-Pay	0.85 (0.63-1.14)	0.27	0.30	0.73 (0.54-0.98)	0.04	0.24	0.77 (0.58-1.01)	0.06	0.28	0.87 (0.65-1.16)	0.33	0.28	0.73 (0.54-1.00)	0.05	0.26
Unspecified	0.49 (0.41-0.58)	<.001	0.20	0.57 (0.48-0.69)	<.001	0.20	0.56 (0.47-0.66)	<.001	0.22	0.65 (0.55-0.77)	<.001	0.22	0.56 (0.47-0.66)	<.001	0.21
**Cancer Category**															
Prostate	Ref		0.14	Ref		0.10	Ref		0.14	Ref		0.14	Ref		0.15
Bladder and ureter	3.17 (2.75-3.65)	<.001	0.34	2.82 (2.40-3.32)	<.001	0.23	1.94 (1.66-2.26)	<.001	0.25	2.41 (2.08-2.79)	<.001	0.29	1.37 (1.16-1.62)	<.001	0.19
Bone soft tissue and sarcoma	2.89 (2.49-3.36)	<.001	0.32	5.20 (4.47-6.05)	<.001	0.36	4.02 (3.48-4.64)	<.001	0.40	2.29 (1.97-2.67)	<.001	0.28	2.72 (2.33-3.17)	<.001	0.32
Breast	2.65 (2.35-2.99)	<.001	0.31	3.02 (2.64-3.47)	<.001	0.25	2.92 (2.59-3.30)	<.001	0.33	1.91 (1.68-2.17)	<.001	0.24	2.61 (2.31-2.95)	<.001	0.31
CNS	1.33 (1.15-1.54)	<.001	0.18	8.06 (7.03-9.25)	<.001	0.47	3.84 (3.38-4.36)	<.001	0.39	1.61 (1.39-1.85)	<.001	0.21	10.14 (8.90-11.55)	<.001	0.63
Colorectal	3.33 (2.90-3.82)	<.001	0.36	3.78 (3.26-4.39)	<.001	0.29	1.92 (1.66-2.21)	<.001	0.24	3.15 (2.75-3.60)	<.001	0.34	2.02 (1.75-2.34)	<.001	0.26
GI other	2.18 (1.80-2.64)	<.001	0.27	5.14 (4.26-6.20)	<.001	0.36	1.89 (1.55-2.29)	<.001	0.24	3.22 (2.68-3.88)	<.001	0.35	1.81 (1.48-2.22)	<.001	0.24
Gynecologic	1.88 (1.64-2.15)	<.001	0.24	3.21 (2.78-3.72)	<.001	0.26	1.86 (1.62-2.12)	<.001	0.24	2.21 (1.94-2.53)	<.001	0.27	2.29 (2.01-2.61)	<.001	0.28
Head and neck	1.60 (1.34-1.92)	<.001	0.21	4.40 (3.68-5.25)	<.001	0.32	3.95 (3.36-4.64)	<.001	0.40	3.56 (3.04-4.18)	<.001	0.37	2.04 (1.70-2.44)	<.001	0.26
Hematologic	7.02 (6.35-7.76)	<.001	0.54	7.49 (6.68-8.40)	<.001	0.45	4.43 (4.00-4.90)	<.001	0.43	4.20 (3.79-4.65)	<.001	0.41	2.98 (2.69-3.30)	<.001	0.34
Kidney and renal pelvis	1.56 (1.29-1.89)	<.001	0.21	2.98 (2.45-3.63)	<.001	0.24	1.91 (1.59-2.31)	<.001	0.24	2.17 (1.80-2.60)	<.001	0.26	1.65 (1.36-2.01)	<.001	0.22
Liver and bile duct	3.20 (2.82-3.64)	<.001	0.35	4.36 (3.79-5.03)	<.001	0.32	3.55 (3.13-4.03)	<.001	0.37	2.93 (2.58-3.34)	<.001	0.33	2.02 (1.76-2.32)	<.001	0.26
Lung	2.71 (2.27-3.24)	<.001	0.31	4.52 (3.79-5.39)	<.001	0.33	2.35 (1.98-2.79)	<.001	0.28	5.25 (4.48-6.15)	<.001	0.47	1.98 (1.65-2.39)	<.001	0.25
Melanoma	1.95 (1.51-2.50)	<.001	0.24	3.74 (2.91-4.82)	<.001	0.29	2.88 (2.27-3.64)	<.001	0.33	1.94 (1.49-2.52)	<.001	0.24	2.70 (2.11-3.44)	<.001	0.32
Multiple Diagnoses	4.48 (3.61-5.56)	<.001	0.43	5.59 (4.56-6.85)	<.001	0.38	5.34 (4.48-6.36)	<.001	0.47	4.30 (3.51-5.27)	<.001	0.42	2.75 (2.21-3.42)	<.001	0.32
Non melanoma skin	1.93 (1.68-2.22)	<.001	0.24	3.58 (3.09-4.16)	<.001	0.28	3.19 (2.79-3.64)	<.001	0.35	2.56 (2.23-2.95)	<.001	0.30	1.69 (1.46-1.95)	<.001	0.22
Other	2.59 (2.29-2.93)	<.001	0.30	2.53 (2.19-2.92)	<.001	0.22	2.04 (1.80-2.33)	<.001	0.26	2.66 (2.34-3.02)	<.001	0.31	2.26 (1.99-2.57)	<.001	0.28
Pancreatic	3.77 (3.17-4.47)	<.001	0.38	4.37 (3.63-5.26)	<.001	0.32	2.76 (2.31-3.28)	<.001	0.32	2.88 (2.42-3.42)	<.001	0.32	1.12 (0.90-1.40)	0.30	0.16
Unspecified malignant neoplasm	3.79 (3.44-4.17)	<.001	0.39	6.36 (5.68-7.11)	<.001	0.41	4.11 (3.72-4.54)	<.001	0.41	3.95 (3.57-4.36)	<.001	0.40	3.00 (2.72-3.31)	<.001	0.34

© 2025 Chang C et al. *JAMA Network Open.*

eTable 9: Multivariable GEE analysis for documented symptoms across demographic and cancer characteristics

Variables	Pain		Nausea		Vomiting		Fatigue		Constipation	
	OR	p-value	OR	p-value	OR	p-value	OR	p-value	OR	p-value
**Sex**										
Male	Ref		Ref		Ref		Ref		Ref	
Female	1.13 (1.06-1.21)	<.001	1.22 (1.16-1.30)	<.001	1.14 (1.09-1.20)	<.001	1.07 (1.03-1.12)	0.002	1.14 (1.09-1.19)	<.001
Unknown	0.82 (0.26-2.58)	0.74	0.56 (0.20-1.56)	0.27	1.12 (0.42-2.97)	0.82	1.03 (0.29-3.58)	0.97	0.90 (0.27-3.03)	0.87
**Age**										
18-65	Ref		Ref		Ref		Ref		Ref	
>= 65	0.74 (0.68-0.81)	<.001	0.73 (0.67-0.78)	<.001	0.75 (0.70-0.80)	<.001	0.99 (0.94-1.05)	0.79	0.95 (0.89-1.01)	0.11
**Race and Ethnicity**										
White	Ref		Ref		Ref		Ref		Ref	
Asian	0.79 (0.73-0.86)	<.001	0.82 (0.77-0.88)	<.001	0.83 (0.78-0.88)	<.001	1.05 (0.99-1.11)	0.10	1.18 (1.12-1.25)	<.001
Black or African American	0.83 (0.72-0.95)	0.007	0.62 (0.56-0.68)	<.001	0.62 (0.57-0.68)	<.001	0.84 (0.77-0.91)	<.001	1.17 (1.07-1.28)	<.001
Native American or Alaska Native	1.07 (0.73-1.56)	0.73	0.79 (0.60-1.04)	0.10	0.81 (0.64-1.02)	0.08	1.29 (1.03-1.62)	0.03	1.02 (0.80-1.29)	0.87
Native Hawaiian or Other Pacific Islander	1.01 (0.71-1.44)	0.96	0.87 (0.65-1.17)	0.35	0.79 (0.62-1.01)	0.06	1.16 (0.92-1.45)	0.20	1.19 (0.95-1.50)	0.12
Unknown/Declined	0.89 (0.67-1.20)	0.45	0.79 (0.64-0.98)	0.04	0.93 (0.76-1.13)	0.45	0.71 (0.60-0.85)	<.001	0.79 (0.65-0.96)	0.02
Other	1.08 (0.97-1.19)	0.16	0.95 (0.88-1.03)	0.25	0.97 (0.91-1.04)	0.36	0.95 (0.89-1.01)	0.08	1.07 (1.00-1.14)	0.04
**Insurance**										
Private	Ref		Ref		Ref		Ref		Ref	
Medicaid	1.17 (1.06-1.30)	0.002	0.91 (0.85-0.99)	0.02	1.00 (0.94-1.07)	0.98	0.91 (0.86-0.97)	0.002	1.03 (0.97-1.09)	0.35
Medicare	0.92 (0.84-1.01)	0.08	0.84 (0.78-0.91)	<.001	0.88 (0.82-0.93)	<.001	0.93 (0.87-0.98)	0.010	1.01 (0.95-1.08)	0.77
Other	1.12 (0.51-2.47)	0.77	0.87 (0.50-1.50)	0.61	1.25 (0.72-2.16)	0.43	1.11 (0.71-1.74)	0.64	0.59 (0.37-0.94)	0.03
Uninsured/Self-Pay	0.77 (0.51-1.15)	0.21	0.87 (0.61-1.25)	0.45	1.03 (0.74-1.43)	0.87	0.90 (0.68-1.18)	0.43	0.78 (0.58-1.05)	0.11
Unspecified	0.71 (0.56-0.90)	0.005	0.59 (0.50-0.71)	<.001	0.75 (0.64-0.89)	<.001	0.71 (0.61-0.82)	<.001	0.72 (0.61-0.84)	<.001
**Cancer Category**										
Prostate	Ref		Ref		Ref		Ref		Ref	
Bladder and ureter	0.82 (0.65-1.05)	0.12	0.66 (0.56-0.77)	<.001	0.60 (0.52-0.70)	<.001	1.30 (1.15-1.47)	<.001	3.25 (2.83-3.73)	<.001
Bone soft tissue and sarcoma	1.05 (0.78-1.40)	0.76	0.72 (0.60-0.86)	<.001	0.61 (0.53-0.72)	<.001	1.50 (1.32-1.70)	<.001	3.04 (2.63-3.53)	<.001
Breast	0.49 (0.39-0.60)	<.001	0.50 (0.43-0.58)	<.001	0.45 (0.40-0.52)	<.001	1.12 (1.00-1.25)	0.04	2.70 (2.38-3.05)	<.001
CNS	0.21 (0.17-0.25)	<.001	0.46 (0.40-0.54)	<.001	0.35 (0.30-0.40)	<.001	1.43 (1.28-1.60)	<.001	2.22 (1.95-2.53)	<.001
Colorectal	0.62 (0.49-0.79)	<.001	0.84 (0.71-1.01)	0.06	0.64 (0.55-0.75)	<.001	1.61 (1.43-1.82)	<.001	4.08 (3.58-4.66)	<.001
GI other	0.63 (0.46-0.87)	0.006	0.80 (0.63-1.00)	0.05	0.73 (0.60-0.89)	0.002	1.59 (1.35-1.88)	<.001	2.91 (2.42-3.51)	<.001
Gynecologic	0.88 (0.68-1.13)	0.32	0.85 (0.72-1.01)	0.07	0.75 (0.65-0.88)	<.001	1.37 (1.22-1.54)	<.001	2.51 (2.19-2.87)	<.001
Head and neck	0.82 (0.60-1.11)	0.20	0.84 (0.68-1.03)	0.10	0.64 (0.54-0.77)	<.001	1.29 (1.12-1.49)	<.001	3.88 (3.31-4.56)	<.001
Hematologic	0.24 (0.20-0.28)	<.001	0.52 (0.46-0.58)	<.001	0.26 (0.24-0.29)	<.001	1.70 (1.57-1.85)	<.001	3.29 (2.97-3.63)	<.001
Kidney and renal pelvis	0.60 (0.45-0.80)	<.001	0.53 (0.44-0.65)	<.001	0.46 (0.38-0.55)	<.001	1.02 (0.87-1.19)	0.85	1.48 (1.22-1.79)	<.001
Liver and bile duct	0.26 (0.22-0.32)	<.001	0.37 (0.32-0.42)	<.001	0.26 (0.23-0.30)	<.001	1.26 (1.12-1.41)	<.001	2.27 (2.00-2.58)	<.001
Lung	0.48 (0.37-0.62)	<.001	0.50 (0.42-0.59)	<.001	0.41 (0.34-0.48)	<.001	1.32 (1.14-1.52)	<.001	2.26 (1.91-2.69)	<.001
Melanoma	0.54 (0.36-0.79)	0.002	0.41 (0.32-0.54)	<.001	0.37 (0.30-0.47)	<.001	0.85 (0.68-1.06)	0.15	2.02 (1.59-2.57)	<.001
Multiple Diagnoses	0.48 (0.35-0.67)	<.001	0.59 (0.45-0.77)	<.001	0.44 (0.35-0.55)	<.001	1.65 (1.35-2.02)	<.001	4.52 (3.66-5.58)	<.001
Non melanoma skin	0.25 (0.21-0.31)	<.001	0.21 (0.18-0.24)	<.001	0.19 (0.16-0.21)	<.001	0.73 (0.65-0.82)	<.001	1.48 (1.29-1.71)	<.001
Other	0.68 (0.54-0.84)	<.001	0.67 (0.58-0.77)	<.001	0.57 (0.50-0.65)	<.001	1.23 (1.11-1.37)	<.001	2.34 (2.07-2.64)	<.001
Pancreatic	0.94 (0.68-1.31)	0.74	1.24 (0.97-1.58)	0.08	0.89 (0.73-1.08)	0.25	1.78 (1.51-2.09)	<.001	3.44 (2.90-4.09)	<.001
Unspecified malignant neoplasm	0.61 (0.52-0.72)	<.001	0.71 (0.64-0.80)	<.001	0.46 (0.41-0.51)	<.001	2.06 (1.90-2.23)	<.001	4.57 (4.15-5.04)	<.001

© 2025 Chang C et al. *JAMA Network Open.*

Variables	Fever		Generalized muscle weakness		Severity Edema		Dyspnea		Headache	
	OR	p-value	OR	p-value	OR	p-value	OR	p-value	OR	p-value
**Sex**										
Male	Ref		Ref		Ref		Ref		Ref	
Female	0.99 (0.94-1.03)	0.58	0.98 (0.94-1.03)	0.43	1.09 (1.04-1.14)	<.001	1.05 (1.00-1.10)	0.05	1.36 (1.30-1.43)	<.001
Unknown	0.90 (0.30-2.71)	0.85	0.82 (0.24-2.85)	0.76	0.13 (0.02-0.93)	0.04	0.62 (0.20-1.86)	0.39	0.67 (0.17-2.64)	0.57
**Age**										
18-65	Ref		Ref		Ref		Ref		Ref	
>= 65	0.88 (0.83-0.93)	<.001	1.19 (1.11-1.27)	<.001	1.04 (0.98-1.11)	0.18	1.15 (1.08-1.22)	<.001	0.80 (0.75-0.85)	<.001
**Race and Ethnicity**										
White	Ref		Ref		Ref		Ref		Ref	
Asian	1.27 (1.20-1.35)	<.001	1.29 (1.21-1.36)	<.001	1.13 (1.07-1.20)	<.001	1.24 (1.17-1.31)	<.001	1.00 (0.94-1.06)	0.95
Black or African American	1.01 (0.92-1.11)	0.82	1.38 (1.26-1.51)	<.001	1.25 (1.15-1.37)	<.001	1.30 (1.19-1.42)	<.001	1.00 (0.91-1.11)	0.94
Native American or Alaska Native	1.12 (0.88-1.43)	0.36	1.18 (0.92-1.51)	0.18	0.97 (0.77-1.22)	0.80	1.11 (0.88-1.41)	0.37	1.28 (1.02-1.61)	0.03
Native Hawaiian or Other Pacific Islander	1.28 (1.03-1.59)	0.03	1.24 (0.99-1.56)	0.06	1.34 (1.06-1.69)	0.01	1.21 (0.96-1.52)	0.11	1.11 (0.86-1.42)	0.43
Unknown/Declined	0.67 (0.55-0.83)	<.001	0.96 (0.79-1.17)	0.68	0.76 (0.61-0.93)	0.009	0.96 (0.79-1.16)	0.67	0.92 (0.75-1.12)	0.39
Other	1.12 (1.05-1.20)	<.001	1.14 (1.07-1.22)	<.001	0.99 (0.93-1.06)	0.80	1.05 (0.99-1.12)	0.12	1.02 (0.96-1.09)	0.54
**Insurance**										
Private	Ref		Ref		Ref		Ref		Ref	
Medicaid	1.05 (0.99-1.12)	0.11	1.13 (1.06-1.20)	<.001	1.05 (0.99-1.12)	0.10	1.06 (1.00-1.13)	0.05	1.01 (0.95-1.08)	0.66
Medicare	0.90 (0.84-0.96)	0.001	1.12 (1.05-1.20)	<.001	1.02 (0.96-1.09)	0.46	1.04 (0.97-1.10)	0.25	0.89 (0.83-0.95)	<.001
Other	1.20 (0.76-1.89)	0.43	1.08 (0.68-1.71)	0.74	0.65 (0.41-1.02)	0.06	1.16 (0.75-1.78)	0.50	1.13 (0.72-1.76)	0.60
Uninsured/Self-Pay	1.04 (0.77-1.40)	0.80	0.74 (0.53-1.02)	0.06	0.85 (0.64-1.13)	0.27	1.01 (0.75-1.36)	0.93	0.64 (0.46-0.89)	0.008
Unspecified	0.64 (0.53-0.77)	<.001	0.85 (0.70-1.03)	0.09	0.80 (0.67-0.95)	0.01	0.83 (0.70-0.99)	0.04	0.77 (0.64-0.93)	0.006
**Cancer Category**										
Prostate	Ref		Ref		Ref		Ref		Ref	
Bladder and ureter	3.21 (2.78-3.70)	<.001	2.68 (2.28-3.15)	<.001	1.86 (1.59-2.17)	<.001	2.30 (1.99-2.67)	<.001	1.31 (1.10-1.55)	0.002
Bone soft tissue and sarcoma	2.62 (2.25-3.05)	<.001	5.17 (4.43-6.03)	<.001	3.79 (3.28-4.39)	<.001	2.23 (1.91-2.60)	<.001	2.17 (1.85-2.53)	<.001
Breast	2.38 (2.09-2.71)	<.001	3.16 (2.73-3.65)	<.001	2.66 (2.34-3.03)	<.001	1.85 (1.62-2.13)	<.001	1.72 (1.51-1.97)	<.001
CNS	1.19 (1.03-1.38)	0.02	8.73 (7.59-10.03)	<.001	3.74 (3.29-4.26)	<.001	1.65 (1.42-1.90)	<.001	8.01 (7.01-9.15)	<.001
Colorectal	3.06 (2.66-3.51)	<.001	3.81 (3.27-4.42)	<.001	1.83 (1.58-2.11)	<.001	3.10 (2.70-3.55)	<.001	1.62 (1.40-1.87)	<.001
GI other	1.99 (1.64-2.42)	<.001	4.85 (4.01-5.86)	<.001	1.77 (1.45-2.15)	<.001	3.03 (2.51-3.65)	<.001	1.56 (1.27-1.92)	<.001
Gynecologic	1.70 (1.47-1.96)	<.001	3.22 (2.77-3.76)	<.001	1.67 (1.45-1.93)	<.001	2.10 (1.82-2.43)	<.001	1.52 (1.32-1.76)	<.001
Head and neck	1.49 (1.25-1.79)	<.001	4.19 (3.50-5.01)	<.001	3.71 (3.15-4.36)	<.001	3.38 (2.88-3.98)	<.001	1.75 (1.46-2.09)	<.001
Hematologic	6.39 (5.77-7.09)	<.001	7.48 (6.66-8.41)	<.001	4.21 (3.80-4.67)	<.001	4.12 (3.71-4.57)	<.001	2.41 (2.17-2.68)	<.001
Kidney and renal pelvis	1.45 (1.19-1.76)	<.001	2.91 (2.39-3.55)	<.001	1.80 (1.50-2.18)	<.001	2.09 (1.73-2.51)	<.001	1.37 (1.13-1.67)	0.002
Liver and bile duct	2.86 (2.51-3.26)	<.001	4.12 (3.57-4.75)	<.001	3.34 (2.94-3.80)	<.001	2.78 (2.44-3.17)	<.001	1.71 (1.49-1.97)	<.001
Lung	2.61 (2.18-3.12)	<.001	4.13 (3.46-4.93)	<.001	2.13 (1.79-2.55)	<.001	4.77 (4.06-5.61)	<.001	1.71 (1.41-2.06)	<.001
Melanoma	1.92 (1.49-2.47)	<.001	3.97 (3.08-5.13)	<.001	2.81 (2.22-3.57)	<.001	1.98 (1.51-2.58)	<.001	2.18 (1.70-2.79)	<.001
Multiple Diagnoses	4.25 (3.43-5.28)	<.001	5.44 (4.45-6.65)	<.001	4.98 (4.17-5.94)	<.001	4.10 (3.35-5.02)	<.001	2.32 (1.86-2.90)	<.001
Non melanoma skin	1.94 (1.69-2.24)	<.001	3.59 (3.10-4.17)	<.001	3.07 (2.68-3.51)	<.001	2.53 (2.19-2.91)	<.001	1.48 (1.28-1.71)	<.001
Other	2.31 (2.03-2.63)	<.001	2.61 (2.26-3.03)	<.001	1.94 (1.70-2.22)	<.001	2.64 (2.32-3.00)	<.001	1.69 (1.47-1.93)	<.001
Pancreatic	3.55 (2.98-4.22)	<.001	4.21 (3.49-5.07)	<.001	2.57 (2.16-3.07)	<.001	2.72 (2.28-3.24)	<.001	0.93 (0.74-1.15)	0.49
Unspecified malignant neoplasm	3.43 (3.10-3.79)	<.001	6.32 (5.63-7.09)	<.001	3.84 (3.47-4.26)	<.001	3.81 (3.44-4.23)	<.001	2.35 (2.12-2.61)	<.001

© 2025 Chang C et al. *JAMA Network Open.*
