# Supplementary material for: Patterns in Symptoms Preceding Acute Care in Patients With Cancer
Source: JAMA Netw Open. 2025 Apr 22;8(4):e256366. doi: 10.1001/jamanetworkopen.2025.6366 (PMC12015675; doi:10.1001/jamanetworkopen.2025.6366)
Supplement: Supplement 2. — Data Sharing Statement [file jamanetwopen-e256366-s002.pdf]

Data Sharing Statement

Chang. Patterns in Symptoms Preceding Acute Care in Patients With Cancer. *JAMA Netw Open*. Published April 22, 2025. doi:10.1001/jamanetworkopen.2025.6366

Data
Data available: No

Additional Information
Explanation for why data not available: Sensitivity of free text clinical data/privacy.
